# Molecular Mechanisms of Dietary Compounds in Cancer Stem Cells from Solid Tumors: Insights into Colorectal, Breast, and Prostate Cancer

**DOI:** 10.3390/ijms26020631

**Published:** 2025-01-13

**Authors:** Alexandru Filippi, Teodora Deculescu-Ioniță, Ariana Hudiță, Oana Baldasici, Bianca Gălățeanu, Maria-Magdalena Mocanu

**Affiliations:** 1 Department of Biochemistry and Biophysics, “Carol Davila” University of Medicine and Pharmacy of Bucharest, 050474 Bucharest, Romania; alexandru.filippi@umfcd.ro; 2 Department of Pharmacognosy, Phytochemistry and Phytotherapy, “Carol Davila” University of Medicine and Pharmacy of Bucharest, 050474 Bucharest, Romania; teodora.deculescu-ionita@umfcd.ro; 3 Department of Biochemistry and Molecular Biology, University of Bucharest, 050095 Bucharest, Romania; ariana.hudita@bio.unibuc.ro (A.H.); bianca.galateanu@bio.unibuc.ro (B.G.); 4 Department of Genetics, Genomics and Experimental Pathology, The Oncology Institute “Prof. Dr. Ion Chiricuță”, 400015 Cluj-Napoca, Romania; oana.baldasici@iocn.ro

**Keywords:** cancer stem cells, diet, colorectal cancer, breast cancer, prostate cancer, epithelial–mesenchymal transition, signaling pathways

## Abstract

Cancer stem cells (CSC) are known to be the main source of tumor relapse, metastasis, or multidrug resistance and the mechanisms to counteract or eradicate them and their activity remain elusive. There are different hypotheses that claim that the origin of CSC might be in regular stem cells (SC) and, due to accumulation of mutations, these normal cells become malignant, or the source of CSC might be in any malignant cell that, under certain environmental circumstances, acquires all the qualities to become CSC. Multiple studies indicate that lifestyle and diet might represent a source of wellbeing that can prevent and ameliorate the malignant phenotype of CSC. In this review, after a brief introduction to SC and CSC, we analyze the effects of phenolic and non-phenolic dietary compounds and we highlight the molecular mechanisms that are shown to link diets to CSC activation in colon, breast, and prostate cancer. We focus the analysis on specific markers such as sphere formation, CD surface markers, epithelial–mesenchymal transition (EMT), Oct4, Nanog, Sox2, and aldehyde dehydrogenase 1 (ALDH1) and on the major signaling pathways such as PI3K/Akt/mTOR, NF-κB, Notch, Hedgehog, and Wnt/β-catenin in CSC. In conclusion, a better understanding of how bioactive compounds in our diets influence the dynamics of CSC can raise valuable awareness towards reducing cancer risk.

## 1. Introduction

Cancer relapse, metastasis, or tumor heterogeneity are processes proposed to have been originated in very few undifferentiated cells with a high capacity for self-renewal, known as cancer stem cells. Up to now, several very well-documented papers have greatly explained the characteristics of CSC, their functionality, or niche and have shown that their fate is regulated by extracellular and intracellular signals, transduction pathways, or transcriptional factors [1,2,3]. Nevertheless, in the human body, the stemness of the cells is a known feature due to the fact that embryo, epidermis, intestinal, hematopoietic, or germ stem cells are responsible for differentiation in somatic and germ lines [4]. To date, there have been several models proposed for the role of CSC in carcinogenesis and the most accepted of which are the hierarchical and the stochastic models. The main differences between these two models of CSC in carcinogenesis consist of the following: (i) in the hierarchical model, the initial cell is a normal stem cell that undergoes oncogenic mutations, while in the stochastic model, the initial cell is a normal epithelial cell (or other type of cell) that undergoes oncogenic mutations and acquires stem-like features [5]; (ii) the hierarchical model implicates asymmetrical division with the formation of one self-renewal cell and one progenitor cell that will generate differentiated cancer cells, while the second one does not imply asymmetrical division [6]; (iii) in the hierarchical model, only a set of cells are able to propagate tumorigenesis, while in the stochastic model, every cell from the tumor can facilitate malignant progression [5]. However, these two models are not mutually exclusive and a very interesting phenomenon named cellular plasticity is able to connect the two proposed models of carcinogenesis [2,5]. In addition to these powerful characteristics that offer high adaptability to the environment, CSC are quiescent cells, a feature that make them able to escape radio- and chemotherapeutic approaches or the immune surveillance which, in turn, will conduct to tumor relapse [7,8].

One of the major aims of humanity is the eradication of malignant transformation and this can be possible when the known causes of the disease are removed or all the steps that prevent the apparition of the illness are taken. Regarding prevention, lifestyle is critical and eight hours rest every night, physical exercise, stress management, an optimal diet, education, and an equilibrated life–work balance can result in many health benefits [9,10,11,12,13]. This review focuses on diet; for instance, it has already been reported that epigallocatechin-3-gallate (EGCG), a polyphenol from green tea, can reduce the growth of CSC in colon samples [14,15]. At the same time, walnuts rich in bioactive agents such as polyunsaturated fatty acids (PUFA), tocopherols, or ellagic acid (EA) can suppress CSC growth in colon samples [16]. One of the major advantages of the use of dietary factors in reducing CSC levels is that all these active compounds administrated in optimal doses do not have side effects on the human body.

In this study, we focused on the effects of plant-derived dietary compounds on the molecular mechanisms in CSC originating in three types of solid tumors: colorectal, breast, and prostate carcinoma.

## 2. Stem Cells

### 2.1. General Characteristics and Markers of Stem Cells

Adult SC are located in specialized microenvironments within tissues that maintain and regulate their behavior, called stem cell niches. SC coexist in the niche with various cell types, such as fibroblasts and macrophages. Fibroblasts secrete an extracellular matrix with a particular composition, responsible for signaling both in direct interactions with the integrins expressed by SC and indirect through the retention of specific soluble molecules and the promotion of their binding to the respective receptors on SC [17], and macrophages drive stem cell activation and proliferation through the cytokines they secrete [18]. The microenvironment influences SC so significantly that, upon depletion, progenitor or even adult cells within the niche can be reprogramed into stem cells, a process known as cellular plasticity [19].

One of the best described and anatomically distinct stem cell niches is the intestinal stem cell niche located at the base of intestinal/colonic crypts. Here, intestinal stem cells (ICS) are interspersed with Paneth cells, while progenitor cells resulting from the asymmetrical division of stem cells can be found higher up on the walls of the crypt, in the transit amplifying region. These progenitor cells are short lived and after four–five divisions give rise to different types of terminally differentiated cells, such as the epithelial cells that move further up to replace shed cells in the villi, or Paneth cells that remain at the bottom of the crypt, in the stem cell niche [20]. In other tissues, the stem cell niche is less well understood. For example, in the prostate, it has been long known that SC are grouped in the basal compartment of the proximal duct [21] or possibly both in the basal compartment as well as the luminal compartment of the proximal duct [22]; however, more recent data show that most luminal cells, not just a rare subpopulation of SC, are able to proliferate and regenerate the androgen-ablated prostate [23]. In a similar example, in the mammary tissue, mammary SC can be found in the basal compartment and closely communicate with macrophages and stromal cells who provide the signals needed to maintain their stemness [24]. Mammary SC are bipotent: they can give rise both to luminal and basal/ myoepithelial cells [25]

In the past decades, in order to set up standardized protocols for purifying and analyzing stem cells, intensive studies were performed towards the identification of stem cell markers (Table 1).

Thus, we know that ISC steadily express markers CD133 [28], leucine-rich repeat containing G protein coupled receptor 5 (Lgr5), telomerase reverse transcriptase (Tert), HOP Homeobox (Hopx), leucine-rich repeats and immunoglobulin-like domains protein 1 (Lrig1) [33], Olfactomedin 4 (OLFM4) [34], Polycomb Complex Protein Bmi1 [33,36], and aldehyde dehydrogenases class I (ALDH1) [26], among others; mammary SC markers include ALDH1 [27], CD29, CD24 [30,32], CD49f [32], CD133 [29], and Sca-1 [35]; and prostate SCs express CD133, Stem cells antigen-1 (Sca1), B-cell lymphoma 2 (Bcl-2), keratins K5/K14, p27, CD44, and CD49f [22,31]. As the above examples demonstrate, SC from different tissues can express widely different markers, though an overlap can still be observed (see [37] for a comprehensive review on the stem cell markers in other tissues such as the nervous system, heart, pancreas, and liver).

It is to be noted that due to cellular plasticity, some authors question even the possibility of defining tissue-specific SC based on an absolute set of markers, as stem cells derived from progenitor or terminally differentiated cells might keep some traits of their parent cells [38].

Thus, after tissue-specific SC have been successfully analyzed in most of the organs, the research has shifted more and more towards the metabolic functions and molecular pathways in SC, which could provide more insight in the function of these cells and also provide therapeutic targets.

### 2.2. Molecular Pathways in Stem Cells

The ability of stem cells to proliferate, self-renew, and differentiate in specific cell types, in other words, their stemness, is regulated by the activation of key signaling pathways such as Wnt, Notch, and bone morphogenetic protein (BMP). Signaling through these pathways is highly interconnected [39] and leads to the regulation of transcription factors controlling the expression of genes involved in proliferation [40] and differentiation [41] in the case of Wnt, differentiation and apoptosis for BMP [42,43], and proliferation, survival, and the prevention of differentiation for Notch [44].

Wnt ligands bind to Frizzled receptors expressed by the stem cells and also to the Lrp5/6 co-receptor, forming a complex that induces a conformational change that activates the phosphorylation of Lrp, which inhibits glycogen synthase kinase 3 (GSK3) and binds Axin. In the absence of Wnt signaling, Axin and GSK3, together with adenomatous polyposis coli (APC), form a complex that phosphorylates β-catenin, targeting it for degradation. Wnt signaling inhibits this complex, allowing β-catenin to accumulate and enter the nucleus, where it interacts with T cell factor (TCF)/lymphoid enhancer factor (LEF) transcription factors to regulate gene expression [45,46].

The Notch pathway is initiated in SC through delta-like ligands (DLL) 1, 3, 4, Jagged-1 (JAG1), and Jagged-2 (JAG2) [47], which induce the release of the intracellular domain of Notch receptors (NICD) through proteolytic cleavages [48]. NICD translocate to the nucleus where it activates the target gene transcription of hairy/enhancer of split 1 (Hes1). In turn, Hes1 represses the cell-cycle regulators p27Kip1 and p57Kip2 [49]. The Notch pathway is also regulated by other factors in the SC niche. Macrophage-secreted IL6 inhibits, TGF-β1 positively regulates Notch signaling, and shear stress indirectly activates Notch by the VEGF-induced secretion of DLL4 [50].

While Wnt and Notch signaling confer the SC different characters, with Wnt enhancing proliferation and survival and Notch preventing differentiation, their operation is interdependent, as the Wnt-mediated maintenance of undifferentiated SC requires intact Notch signaling [51] and Notch signaling can be increased through Wnt signaling: Notch1 is under the control of the E2F1, which in turn is controlled by the Wnt target p21 through the p21-DREAM/MMB/Rb-E2F1 pathway [52].

It was shown that SC from glandular organs, such as the prostate or the mammary gland, require EGFR in addition to Wnt and Notch signaling to maintain their multipotency and sufficient levels of cell proliferation [53]. The same study identified the role of TNFα in restricting the multipotency of basal SC [53].

Cells forming the SC niche provide stimuli for the activation of all of these pathways. It has been shown that, in intestinal crypts, Paneth cells express EGF, TGF-a, Wnt3, and the Notch ligand Dll4, and as such, only cells in close proximity to Paneth cells can maintain their stemness, or gain it—as is the case for newly formed SC from more differentiated cells [54].

BMP signaling was first described as proteins involved in bone and cartilage formation; however, their functions extend to other tissues, including kidney, lung, and intestines [55]. In the canonical pathway, BMP signaling leads to the formation of the SMAD complex which translocate into the nucleus to regulate the transcription of target genes. The non-canonical pathway involves the activation of Runx2 and NF-κB transcription factors downstream of MAPK [56]. In SC, BMP signaling promotes differentiation and cellular lineage commitment, and regulates apoptosis, thereby maintaining tissue homeostasis and proper tissue development [42].

## 3. Cancer Stem Cells

### 3.1. General Characteristics and Markers of CSC

It has long been hypothesized that since in most tissues SC are the only cells that persist for long enough to acquire sufficient genetic alterations for developing cancers, these cells, and not the terminally differentiated cells, must be the source of cancer [57]. Regardless of their origin, CSC are the functional analog of SC, representing a subpopulation of cancer cells with the abilities of self-renewal and differentiation, which can drive tumor growth and recurrence. Similarly to SC, CSC can have high plasticity, with tumor cells being able to transition between different phenotypes such as between stem cell, basal, or luminal cells in the case of breast cancer [58].

The tumor microenvironment is made up of a multitude of cells besides cancer cells, including cancer-associated fibroblasts (CAF), stromal myofibroblasts, endothelial cells, diverse immune cells including tumor-associated macrophages (TAM). Some of these cells were shown to have complex relationships with CSC and are considered to make up the CSC niche. Some CAF might differentiate from CSC and are thought to help in preserving their stemness and also promote invasion and disease progression by the secretion of CXC motif chemokine 12 (CXCL12), transforming growth factor β1 (TGFβ1), platelet-derived growth factor α (PDGFα) [59], IL6, and IL8 [60]. TAM too were found to promote CSC function by the secretion of IL6 [61] and by juxtacrine effects mediated through CD90 [62] and, moreover, CSC might also be one of their sources besides infiltrated monocytes [63]. These interactions are an active topic of research as CSC are believed to be drivers of treatment resistance [64] and cancer relapse [65], and so, disrupting the CSC niche might prove a good therapeutical strategy.

To help isolate and characterize CSC, multiple markers were described, some of them being widely expressed in CSC from many source tissues. CSC from different tumors show different patterns of expressed markers; however, a greater overlap of the markers can be seen between CSC compared with those in SC (Table 2). Thus, for example, CSC from breast cancer express CD44, CD49f, CD133, ALDH1 [66,67], and Sox2 [68]; prostate CSC are known to express CD133 [69], CD44, integrin α2β1 [70], CD49f [71], ALDH1, EZH2, and SOX2 [72]; while colon CSC express CD133 [73], CD44 [74], CD49f [75], and ALDH1 [26], showing an overlap in the CD133, CD44, CD49f, and ALDH1 markers. CD133 is found in most CSC and is thought to preserve stemness through as yet unknown molecular mechanisms [76]. CD44, a receptor for the glycosaminoglycan hyaluronan in the ECM, is not just a marker found on most SCs and CSC but also has known functions in the induction of EMT and protection against reactive oxygen species (ROS) [77]. Another marker expressed in a myriad of different cancers, ALDH1, might prove a useful therapeutic target due to its roles in modulating MYC, VEGF, and Wnt/β-catenin signaling pathways, and in conferring protection from ROS [78].

### 3.2. Molecular Pathways in CSC

The molecular pathways that confer SC their self-renewal and differentiation abilities are also active in CSC, and, in addition to the normal functions, are associated with metastasis, cell growth, and angiogenesis [79].

One of the most frequent pathways activated in CSC is the Wnt pathway. However, its activation is not seen in all cancers and the mechanism that leads to Wnt activation can also vary. For example, in colorectal cancers, loss-of-function mutations of APC are one of the main causes that lead to malignant phenotypes. The loss of APC abrogates β-catenin destruction, leading to its accumulation, mimicking the constitutive activation of Wnt ligand-mediated signaling [80]. In about 10% of colorectal cancers, the activation of the Wnt pathway follows a different route, through mutations in R-spondins [81]. In another 18% of colorectal cancers, Wnt activation is due to Ring Finger Protein 43 (RNF43) mutations, a protein that usually negatively regulates this pathway [82].

Another pathway active in CSC is the Hedgehog pathway, essential in embryonic development, which, in adult organs, contributes to organ homeostasis. This pathway is initiated by the binding of Hedgehog ligands to Patched (PTCH), leading to the activation of Smoothened (SMO), and of Gli family zinc finger protein (Gli) transcription factors that drive the expression of target genes involved in proliferation and survival [83]. While this pathway is not usually active in benign pancreatic cells, in prostate CSC, it is a prerequisite for metastasis [84] and, in mammary CSC, functions to help CSC self-renewal [85].

The Notch signaling pathway plays a critical role in regulating cell fate, proliferation, and differentiation in normal stem cells. When Notch ligands (Delta or Jagged) bind to Notch receptors, proteolytic cleavages release the Notch intracellular domain (NICD), which translocate to the nucleus and controls gene expression related to stem cell maintenance and differentiation [86]. In cancer, Notch signaling is often aberrantly expressed and can function as either an oncogene or a tumor suppressor, depending on the context and interaction with pathways like Wnt [87]. In breast cancer, Notch 1 and Notch 4 promote CSC self-renewal and the formation of metastatic niches [88,89]. In colorectal cancer, Notch signaling is essential for CSC self-renewal and inhibits differentiation and apoptosis, contributing to tumor growth. Notch is also linked to epithelial-to-mesenchymal transition (EMT), which enhances metastatic potential [90,91]. Endothelial cells can further stimulate Notch signaling by secreting Jagged 1, promoting CSC traits and resistance to chemotherapy [92].

As discussed above, the BMP pathway is activated in a myriad of SC and promotes differentiation. Similarly, in CSC, the activation of the BMP pathway generally promotes differentiation and reduces their stemness, thereby inhibiting tumor progression. Conversely, the loss or inhibition of BMP signaling can lead to the increased aggressiveness of cancer by allowing CSC to maintain their undifferentiated, tumorigenic state [93].

CSC were shown to be resistant to radiotherapy due to the inducement of lower ROS levels in these cells as result of higher ROS scavenger activity [94]. This includes the activity of ALDH1, which, during retinoic acid production, reduces ROS levels by detoxifying harmful aldehydes produced during oxidative stress [95]. Moreover, while hypoxic conditions in the tumor microenvironment can promote a shift towards glycolysis, many CSC continue to rely on oxidative phosphorylation and utilize enhanced antioxidant mechanisms to manage ROS, supporting their survival and contributing to therapy resistance [96].

## 4. Dietary Compounds and Cancer Stem Cells

### 4.1. General Information About Dietary Active Agents

Based on preclinical and clinical studies, researchers have emphasized the positive effects of certain foods, known as *functional foods*, upon human health. It is widely known that a dietary pattern (regular intake of fruits, vegetables, fish, and whole grain cereals) along with lifestyle (enough sleep, physical activity, and normal body mass index) patterns have a protective effect on cancer onset. Recent research has focused on the importance of natural compounds and functional foods (as part of a balanced diet) in targeting CSC. Preclinical and clinical studies have demonstrated the role of bioactive compounds (curcumin, epigallocatechin gallate, omega-3 fatty acids, resveratrol, sulphoraphane, quercetin, genistein, etc.) in the modulation of different cellular pathways involved in CSC aggressiveness [97,98].

Recent data have shown that diets rich in antioxidants (polyphenols, vitamin C, carotenoids) and bioactive compounds (mainly omega-3 fatty acids with anti-inflammatory effects) from fruits, vegetables, olive oil, spices, and fish were shown to decrease the risk of breast, colorectal, and prostate cancer through (i) the inhibition of DNA damage, (ii) the inhibition of angiogenesis, (iii) neutralizing free radicals, (iv) the reduction of estrogen levels, and (v) the inhibition of cell division [99]. Mediterranean diet-derived phytochemicals have anti-tumorigenic properties upon CSC through (i) the down-regulation of Notch, Wnt/β-catenin, NF-κB, PI3K/Akt, and Akt-mTOR signaling pathways, (ii) the down-regulation of CSC markers (CD133, DLK1, CD44), (iii) the down-regulation of VEGF and EGFR factors, (iv) the down-regulation of certain cytokines (IL-4, IL-6, IL-8), (v) the down-regulation of ALDH1, ALDH3, and (vi) epigenetic alterations (the inhibition of DNA methyltransferases and histone acetyltransferases) [100,101].

Well studied dietary bioactive compounds (phenolic and non-phenolic constituents) and their food sources can be found in Table 3 and these will be further discussed in this paper regarding their molecular mechanisms in cancer stem cells.

### 4.2. Phenolic Compounds: Flavonoids

#### 4.2.1. Flavones

Flavones, such as apigenin and luteolin, are phenolic compounds with a large distribution in fruits and vegetables such as celery, cabbage, Brussels sprouts, sweet peppers, spinach, apples, or grapes [115,116]. Moreover, high concentrations of luteolin and apigenin are also present in Greek mountain tea (aerial parts of *Siderites* sp.) [105] and other spices (rosemary, thyme, peppermint).

Apigenin. Apigenin derivatives, such as scutellarein (6-hydroxy apigenin) and isoscutellarein, are also found in Greek mountain tea [105]. Polyphenol’s influence upon breast cancer stem cells has been intensively studied using triple negative breast cancer cell lines (MDA-MDB-231, MDA-MDB-436, MDA-MDB-435, MDA-MDB-468, HCC3153, BT-549, etc.). It is well known that triple negative breast cancer is the most lethal subtype of breast cancer, due to the lack of expression of hormone receptors and enriched cancer stem cells populations, which contribute to therapeutic failure and recurrence [117]. According to Ying-Wei L. and co-workers (2018), apigenin has anti-tumor effects on triple negative breast cancer cell lines (TNBC) in a dose-dependent manner through (i) a decreased number of migrated cells, (ii) a decreased number of mammospheres in CD44^+^/CD24^−^ subpopulations, and (iii) the inhibition of transcription factors: yes-associated protein 1 (YAP) and transcriptional coactivator with PDZ-binding motif (TAZ). YAP and TAZ are components of the Hippo signaling pathway and promote cellular proliferation, suppress apoptosis, activate the Wnt/β-catenin signaling pathway, and promote angiogenesis and EMT [117]. Apigenin and kaempferol have shown promising effects on triple breast cancer stem cells through the down-regulation of sirtuins (SIRT3 and SIRT6), since their over expression is associated with the increased bioactivity of CSC [118]. In vitro studies regarding the effect of *Annurca* sp. apple polyphenol extract on triple negative breast cancer cell lines have shown promising results. Polyphenols from *Annurca* sp. apples, a southern Italian variety, were able to inhibit matrix metalloproteinases (MMP-2 and MMP-9) and EMT, down-regulate Smad signaling (crucial for cancer progression), and decrease levels of NF-κB. [119]. Apigenin derivatives, mainly scutellarein, are promising agents targeting breast cancer stem cells. Research regarding their anticancer effect on MDA-MDB-231 and MDA-MDB-361 breast cancer cell lines and preclinical studies revealed a significant reduction in colony formation and mammosphere number, along with the down-regulation of several proteins (CD44, cyclin D1, c-Myc, NF-κB) and signal pathways (mTOR-PI3K/AKT) [120]. Furthermore, scutellarein suppresses the metastasis of triple-negative breast cancer through the down-regulation of G-CSF (granulocyte colony stimulating factor) and TNFR2 receptors, essential for TNF-α production [121]. A recent study has shown that apigenin has anti-tumor effects on prostate cancer stem cells isolated from PC3 and LNCaP cell lines, through the up-regulation of p21, p27 (cyclin-dependent kinase inhibitor), Bax proteins, and caspases-3 and-8 along with the down-regulation of NF-κB, poly (ADP-ribose) polymerases (PARP), and phosphor-p38 (p-p38). It is well known that PARP enzymes are essential for DNA repair and the depletion of NAD^+^/ATP, for apoptotic death, whilst p-p38, a member of the mitogen-activated protein kinase (MAPK) family, is important in stemness [122]. In addition, apigenin’s effect upon prostate cancer stem cells was enhanced by midkine silencing and docetaxel treatment. Midkine is considered a growth factor that promotes cell survival and proliferation in different cells, not only prostate ones [122].

Luteolin. Luteolin inhibits breast cancer stem cells both in vitro and in preclinical studies due to the inhibition of VEGF and the suppression of angiogenesis, the inhibition of medroxyprogesterone acetate stem-cell like properties of breast cancer cells, and the down-regulation of mammosphere formation [123]. Luteolin was found to inhibit the stemness capacity of MDA-MDB-231 cells thorough the down-regulation of stemness-related proteins (Nanog, OCT4, CD44, ABCG2), antioxidant factors (Sirt-3 and Nrf2), and ALDH1 [124]. Furthermore, luteolin showed promising effects upon prostate cancer stem cells through the down-regulation of matrix metaloproteinase-9 (MMP-9) (which is involved in self-renewal and angiogenesis) and the down-regulation of Sox-2 transcriptional factor (which is essential for stem cells pluripotency), ABCG2 efflux transporter, and the JNK signaling pathway [125].

#### 4.2.2. Flavonols

The consumption of different fruits and vegetables (like onions, peppers, lettuce, parsley, broccoli, cappers, tomatoes, etc.) represent an important source of flavonols (quercetin, kaempferol, myricetin, and isorhamnetin) for the human body.

Quercetin. This flavonol was shown to inhibit the growth of breast cancer stem cells, MCF-7, which are resistant to doxorubicine, through the down-regulation of P-gp expression, Y-box binding protein 1 (YB-1) nuclear protein, and the CD44^+^/CD24^−^ phenotype [126]. Moreover, quercetin enhanced the anti-neoplastic effect of doxorubicin, paclitaxel, and vincristine. It was found that quercetin suppressed breast cancer stem cells (CD44^+^/CD24^−^) derived from the MCF7 cell line through the down-regulation of m-TOR, PI3K, and PI3K-AKT pathways and the decreased expression of Bcl-2 protein and cyclin D1 [127]. Quercetin enhanced the doxorubicine effect in T47D and its CD44^+^/CD24^−^ breast cancer cell line through cell cycle arrest and increased apoptosis [128]. According to Turkekuk K. and co-workers (2023), nanoliposome—quercetin nanoparticles—showed promising effects on CD44^+^ cancer stem cells isolated from PC-3 (human androgen resistant) and LNCaP (androgen-sensitive) prostate cancer cell lines, through the inhibition of Wnt/β-catenin signaling pathways, the down-regulation of N-cadherin, p-ERK, and fibronectin [129]. Recent research has shown the beneficial effect of quercetin on PC-3 prostate cancer cells with stem-like properties (induced by treatment with TGF-β). Quercetin inhibited EMT and down-regulated HIF-1α levels and MMP-9 in prostate cancer cell lines [130]. Midkine’s down-regulation in prostate cancer cells treated with quercetin inhibited stem cell proliferation in a dose-dependent matter by reducing the expression of p38, ABCG2, and NF-κB proteins [131].

Kampferol. Recent work has focused on the positive effect of kaempferol on breast cancer stem cells through the down-regulation of MDR-1, ALDH1, and Nanog proteins [132]. Moreover, the association of kaempferol with verapamil inhibits chemoresistance in breast cancer stem cells through the dysregulation of CD44^+^-NANOG-MDR1 pathways [133].

Myricetin. This is another flavonoidic compound that might be a future candidate for the inhibition of different cancer stem cells due to (i) the regulation of matrix metalloproteinases (MMP-2 and MMP-14), (ii) the inhibition of the STAT3 signaling pathway, (iii) the inhibition of epithelial to mesenchymal transition, (iv) the regulation of VEGF activity, and (v) the regulation of immune and inflammatory factors (NF-κB, COX-2, TNF-α, IL-6) [134].

Isorhamnetin. Co-treatment with isorhamnetin and chloroquine showed positive outcomes on triple negative breast cancer cell lines through the inhibition of autophagy, mitochondrial fission, and apoptosis. Mitochondrial fission and apoptosis have been investigated in relation to dynamin-related protein 1 (Drp1), a protein that regulates mitochondrial fission, and its recruitment to mitochondria is linked with phosporylation, S-nitrosylation or ubiquitination, and calmodulin-dependent protein kinase II (CaMKII), which is crucial for the transmission of calcium signals to regulate different processes [135].

#### 4.2.3. Flavanones

*Citrus* sp. (oranges, grapefruit, lemons, mandarins) fresh pulp and peel are an important source of naringenin, tangeretin, hesperidin, nobiletin, and diosmin.

Hesperidin. This flavanone has shown promising effects on an isolated triple negative metastatic breast cancer cell line in BALB/cfC3H mice (4T1 cells) since it inhibits migration and lamellipodia formation through the down-regulation of MMP-9 and Rac-1 protein. Rac-1 protein, also known as Ras-related C3 botulinum toxin substrate 1, is involved in cell-adhesion, motility, and epithelial differentiation [136]. Molecular docking studies have highlighted hesperidin potential to compete with ATP in the ATP binding site of PI3K and thus the inhibition of the PI3K/AKT signaling pathway in breast cancer stem cells. Hesperidin is able to up-regulate p21 expression, which is a CDK-cyclin complex inhibitor protein involved in cell cycle arrest. Other mechanisms involved in hesperidin effects upon breast cancer stem cells involve the up-regulation of p53 and the down-regulation of ALDH1 [137]. A combination of hesperidin and gallic acid treatment on human colorectal cancer cell line (HT-29) showed a strong inhibition of spheroids and the down-regulation of cancer stem cell marker CD133 [138].

Naringenin. Another flavonoid compound found in *Citrus* fruits, narigenin, might be a future candidate for the inhibition of colorectal cancer stem cells, since it down-regulates several signaling pathways (TGF-β, Notch, MAPK-ERK, PI3kinase/Akt/mTOR, JAK-STAT) and Nrf2 production [139].

Nobiletin. A synergism between nobiletin, a phenolic compound found in *Citrus sinensis* (sweet orange) and xantohumol (found in *Humulus lupuls*—common hop) showed antitumor effects on colorectal cancer stem cells. According to Turdo A. and co-workers’ research (2021), a combination of the above-mentioned compounds with chemotherapeutic agents (5-fluorouracil and oxaliplatin) counteracted the clonogenic potential of cancer stem cells and induced apoptosis [140].

Tangeretin. This flavanone was found to inhibit breast cancer stem cells through the inhibition of colony and mammosphere formation, reduced levels of transcriptional factors (Oct2, Nanaog, Sox2), and the inhibition of STAT3 signaling pathways [141]. Tangeretin also showed promising results on prostate cancer stem cells through the inhibition of AKT/mTOR signaling pathways and EMT [142].

#### 4.2.4. Isoflavones

Genistein. The main isoflavone found in soybean is genistein and it has shown promising effects on breast cancer stem cells through the down-regulation of the Hedgehog signaling pathway [143]. Genistein was found to inhibit the growth of colorectal cancer stem cells in an animal model of carcinogenesis induced with dimethyl hydrazine, through the down-regulation of CD133/CD44 and the inhibition of the Wnt/β-catenin signaling pathway [144]. The association of genistein and myokines (oncostatin, irisin) decreased colony and spheres formation in MCF-7 cells and reduced the expression of cancer stem cell markers (Oct4, Sox2) [145]. Genistein along with phenolcarboxylic acids (hippuric acid), isolated from blueberry extract, showed positive effects on MCF-7/MDA-MDB-231 breast cancer cell lines through the inhibition of mammosphere formation and the inhibition of the PI3K/Akt signaling pathway [146]. In addition, the growth of prostate cancer tumorsphere cells was also inhibited by genistein (in vitro studies) through the down-regulation of CD44 markers and the inhibition of the Hedgehog signaling pathway [147]. Recent published data have emphasized the role of genistein in the down-regulation of prostate stem cell cancer antigen (PSCA). However, its effect was similar to that of luteolin but was much lower compared with quercetin [148].

#### 4.2.5. Flavan-3-ols

Catechin, a phenolic compound found in apples, grapes, red wine, and aronia fruits, and epigallocatechin gallate (EGCG), the main constituent of green tea, are known for their anticancer properties, particularly in targeting cancer stem cells.

Catechin. A walnut extract, rich in phenolic compounds (catechin—137.5 mg/100 g, chlorogenic acid—13.6 mg/100 g, ellagic acid—12.6 mg/100 g, and gallic acid—10 mg/100 g) showed promising results on colorectal cancer stem cells by suppressing stemness markers (Notch 1, DLK1, CD44, CD133) and the down-regulation of Wnt/β-catenin signaling pathway [16]. Moreover, the same extract decreased telomere length in a dose dependent manner through the down-regulation of c-Myc and hTERT (human telomerase reverse transcriptase) in a colon cancer stem model [149]. Recent research on catechin isolated from *Aronia* fruits, through lactic fermentation in the presence of *Lactobacillus rhamnosus*, showed anticancer effects on breast cancer stem cells, by the inhibition of mammosphere formation, decreased STAT3, the down-regulation of ALDH1, and the inhibition of IL-6 secretion in mammospheres [150].

EGCG. This polyphenol from green tea inhibits colorectal cancer stem cells through the down-regulation of the Wnt/β-catenin signaling pathway, the up-regulation of GSK-3β (glycogen synthase kinase 3 beta), which is the key negative regulator of the Wnt signaling pathway [14]. In addition, EGCG enhances 5-fluorouracil (5-FU) chemosensitivity in colorectal cancer stem cells through (i) the down-regulation of the Notch pathway, (ii) the dysregulation of polycomb proteins (Bmi-1, Ezh2, Suz12), (iii) the decreased expression of proto-oncogene gene, c-Myc, and (iv) the up-regulated expression of tumor suppressive microRNAs (miR-34a, miR-145, and miR-200c) [15]. Green tea consumption (1.5 g–2.5 g/day equivalent to 5–10 cups of tea daily) showed positive effects upon the recurrence of metachronous colorectal adenomas in 136 patients (which were removed 1 year before enrolment in the present study). Possible explanations for these results consisted in the inhibition of COX-2 expression and the inhibition of EMT and PI3K/AKT signaling pathway [151]. EGCG showed promising results on prostate cancer stem cells through the inhibition of colony and spheres formation, the inhibition of EMT, the down-regulation of vimentin, Bcl-2, and survivin, and the up-regulation of caspase-3 and apoptosis induction [152].

#### 4.2.6. Ellagitannins

Pomegranate (fruits and seeds) is a rich source of ellagitannins (mainly punicagin and punicalagin), with a wide range of therapeutic effects. It is well known that ellagitannins are metabolized by gut microbiota to ellagic acid and further to urolithins. Recent research has revealed that urolithins (uro-A, uro-B, uro-C) inhibit colonospheres formation and ALDH1 activity in colorectal cancer stem cells. Last but not least, uro-A is a substrate for BCRP protein, thus enhancing 5-FU effects upon colorectal CSC [153]. Pomegranate peel extract (rich in gallic acid, ellagic acid, protocatechuic acid, rutin) showed promising effects in a rat model of colorectal cancer and might act upon cancer stem cells through decreased EMT and MMP-9 inhibition [154]. Pomella, a standardized extract of pomegranate fruits with 2.7% ellagic acid and 37.5% punicalagin, significantly decreased mouse mammary cancer stem cells through the up-regulation of pro-apoptotic enzymes (caspase-3) [155]. Moreover, pomegranate peel extracts inhibited EMT in MDA-MDB-231 triple negative breast cancer cell lines [156]. Positive effects upon breast cancer stem cells are also the consequence of Wnt, JNK1, and JNK2 down-regulation [157]. Machado Cahavas and co-workers (2019) have analyzed the effect of lyophilized pomegranate juice and lyophilized peel aqueous extracts (standardized in ellagic acid—51.58 mg/mL and 138.6 mg/mL, respectively] upon prostate CSCs and emphasized their role in reduced colony formation and the inhibition of the Akt/mTOR/S6K signaling pathway [158].

#### 4.2.7. Anthocyanidins

Anthocyanidins (malvidin, peonidin, cyanidin, and their glycosides) are found in high amounts in strawberries, raspberries, bilberries, blueberries, aronia fruits, apples, plums, and purple potatoes. Charepalli V. et al. (2015) have investigated the influence of anthocyanin-containing purple-fleshed potatoes on colorectal cancer stem cells both in vitro and in preclinical studies. According to their results, alcoholic extracts suppressed sphere formation ability, up-regulated cytochrome c and Bax/Bcl-2 proteins ratios, suppressed the Wnt/β-catenin signaling pathway, and reduced the number of crypts [159]. Recent research has highlighted the importance of polyphenol-enriched blueberry preparation on stemness features in two breast cancer cell lines. The authors analyzed the effect of polyphenols from blueberries on two microRNAs: miR-210, an oncogenic molecule able to maintain the CSC phenotype and induce EMT [160], and miR-145, a tumor suppressor molecule that reduces tumor sphere formation and decreases the levels of CD133, CD44, and OCT4 [161]. The administration of the polyphenols from blueberries down-regulated miR-210 and up-regulated miR-145 in triple negative MDA-MDB-231 and highly tumorigenic and invasive 4T1 breast cancer cell lines [161]. Favorable results on breast cancer stem cells (in vitro and animal studies) were also observed for a blueberry-enriched polyphenolic preparation (by fermentation with *Serratia vacci*), through the down-regulation of IL-6/PI3K/Akt and ERK1/2, the inhibition of mammospheres formation, and the inhibition of lung metastasis [162]. The anticancer effect of the enriched polyphenolic preparation was potent compared with the non-fermented juice, due to a high content of phenolic compounds [162].

### 4.3. Phenolic Compounds: Non-Flavonoids

#### 4.3.1. Phenolcarboxylic Acids

Rosmarinic acid. Rosmarinic acid is the main phenolic compound found in rosemary leaves. According to a recent published paper, other constituents (mainly diterpenes and triterpenes—carnasol, carnosic acid, and 12-methoxy carnosic acid) might have antitumor properties for colorectal cancer stem cells through (i) the inhibition of epithelial to mesenchymal transition, (ii) the inhibition of Wnt1 and Wnt 3, (iii) β-catenin down-regulation, (iv) the inhibition of PI3K/AKT/STAT3 signaling pathways [163]. Recent data have shown that rosmarinic acid decreased breast cancer stem cell viability, up-regulated apoptosis, decreased Bcl-2/Bax proteins ratio, and down-regulated miR-30a-5p. It was shown that the down-regulation of miR-30a-5p reduced its silencing effect on the BCL2L11 gene and increased the expression of Bim (a translation product). Furthermore Bim bound to BCL-2 and favored the apoptosis of MDA-MDB-breast cancer stem cells [164]. The association of rosemary leaves and green tea extracts decreased the viability of CD44 breast CSCs and increased apoptosis [165]. An oregano (*Origanum vulgare* L.) alcoholic extract standardized in phenolic compounds (protocatechuic acid, rosmarinic acid, p-coumaric acid, apigenin, and luteolin glycosides) showed anti-tumor effects upon MCF-7 breast cancer cells by means of decreased stem cell biomarkers (CD44, CD24, ALDH1) and the activation of the apoptotic mitochondrial pathway [166].

Chlorogenic acid, caffeic acid, and cinnamic acid. Valuable sources of these acids are plums, apples, artichoke fruits, and coffee. Caffeic acid, along with trans-cinnamic acid, ferulic acid, and *p*-coumaric acid are found in mountain tea infusions prepared from aerial parts of *Sideritis syriaca* (native to Crete), *Siderites raeseri* (native to Macedonia), and *Siderites scardica* (native to Olymp mountain) [167]. In vitro studies regarding the beneficial effects of cinnamic acid on cancer stem cells revealed it positive effect on HT-29 colorectal CSC (CD44^+^, CD133^+^ populations) with decreased viability and the down-regulation of stemness markers (Oct4, Nanog, ALDH1, ABCB1). Moreover, treatment with cinnamic acid dropped cells resistance to chemotherapeutic agents (5-fluorouracil plus oxaliplatin (FOLFOX) [168]. The influence of green and roasted coffee extracts (rich is chlorogenic, neochlorogenic, and criptoclorogenic acids) on colorectal cancer stem cells have been also investigated. Green coffee extracts contained a higher content of chlorogenic acid compared to roasted coffee extracts. The results were promising, with the down-regulation of the Wnt/β-catenin signaling pathway and E-cadherin, cyclin D1, and β-catenin [169]. According to recent research, caffeic acid inhibits the proliferation, migration, and stemness of the DU-145 prostate cancer cell line, through the down-regulation of EMT and the decreased expression of stemness genes, Nanog and Oct4 [170].

Ferulic acid. This acid extracted from *Ferula foetida* showed promising results upon MDA-MDB-231 triple negative breast cancer cell line, through the inhibition of EMT and increased caspase-3 activity. Moreover, mice inoculated with breast cancer showed a marked decrease in tumor growth and the inhibition of lung metastasis in the presence of ferulic acid [171].

#### 4.3.2. Stilbenes

Among stilbenes, resveratrol, piceatannol, and pterostilbene are mostly found in red wine and red grapes. According to a recent literature review, resveratrol and pterostilbene decreased cancer stem cells viability in several malignancies, including breast and colorectal cancer. Both compounds influence cancer stem cells through various mechanisms: (i) the up-regulation of pro-apoptotic genes DAPK2 and BNIP3, (ii) the inhibition of EMT, (iii) the down-regulation of PI3K/Akt, Wnt/β-catenin, and Hedgehog signaling pathways, (iv) enhanced cell surface expression of death receptor DR4, (v) decreased levels of IL-6, (vi) the down-regulation of EMT key activators (Twist 1, Snail 1), (vii) the suppression of antistress protein GRPT8 which is linked to the Notch signaling pathway, (viii) epigenetic mechanisms—the up-regulation of miR-205, and (ix) increased autophagy by Akt/mTOR suppression and SIRT1/p38 induction [172,173].

Resveratrol. Regarding the role of resveratrol on colorectal cancer stem cells, it is well known that gut microbiota modulates the host immune response. Resveratrol increases the abundances of bacteria that produce short-chain fatty acids, mainly butyrate, with an anti-inflammatory effect upon cancer cells. In addition, butyrate is a strong inhibitor of histone deacetylase (HDAC). In a mouse model of colorectal cancer, resveratrol directly influenced gut microbiota, since it decreased *Proteobacteria* and *Desulfovibrio* and increased *Akkermansia*, *Blautia*, and *Clostridium* [174]. Resveratrol showed positive results on the HCT116 colorectal cell line resistant to 5-FU and treated tumor necrosis beta (TNF-β). TNF-β promote inflammation in the tumor microenvironment and chemoresistance. A subpopulation of CSC was isolated from HCT116 treated with TNF-β (mainly CD44+ and ALDH+ cancer stem cells). Treatment with resveratrol potentiated 5-FU apoptosis, blocked TNF-β-induced NF-κB activation, and down-regulated EMT [175]. Furthermore, the association of resveratrol and a grape seed extract (with a total phenolic content > 85% expressed as gallic acid equivalents, rich in catechin/epicatechin monomers and their oligomers) suppressed the sphere-formation ability of colorectal cancer stem cells (in vitro), down-regulated Wnt pathway proteins (pGSK3β, cyclin D1, c-Myc, β-catenin), increased the Bax/Bcl-2 protein ratio, increased p53 (the genome guardian), and decreased COX-2 activity [176]. The association also showed positive effects in an animal model of colorectal cancer (induced with azoxymethan), through a reduction of crypts containing colon CSC with β-catenin [176]. In a pilot phase I clinical study, with patients diagnosed with colorectal cancer, treatment with a red grape dry extract (GP) (containing resveratrol, 4 μM/kg of dry powder; flavonols, 118 μM/kg; anthocyanins, 700 mg/kg; flavans, 3.9 mg/g—as catechin) at 80 g/day and 120 g/day for 14 days showed promising results, through the decreased expression of colorectal stem cells markers (CD133, LGR5) and the down-regulation of the Wnt signaling pathway. The Wnt inhibitory effect was also observed in normal mucosa, suggesting the role of red grapes and resveratrol in chemoprevention of colorectal cancer [177]. In vitro and in vivo studies revealed that resveratrol inhibits breast cancer stem cells, isolated from the MCF-7 cell line, through decreased mammosphere formation, decreased ALDH1 activity, increased autophagy, and the inhibition of Wnt-signaling pathway [178]. Resveratrol was shown to inhibit MDA-MDB-231 stemness through the inhibition of fatty acid synthase, which induced the up-regulation of pro-apoptotic genes (DAPK2, BNIP3) [179]. In addition, resveratrol inhibits the migration and metastasis of the MDA-MDB-231 cancer cell line by reversing TGF-β1 epithelial to mesenchymal transition, inhibiting the PI3K/AKT signaling pathway, down-regulating MMP-2/MMP-9 enzymes, and down-regulating transcription factors involved in EMT (Snail 1, Slug, Smad3, P-Smad2, P-Smad) [180].

Pterostilbene. A key component of blueberries, 3,5-dimethoxy-4-hydroxystilbene, inhibits breast cancer stem cells through the down-regulation of epithelial to mesenchymal transition, the down-regulation of NF-κB, and decreased levels of tumor-associated macrophages (TAMs). It is well known that TAM cells are important in the generation and maintenance of breast CSCs through the increased expression of EMT modulators (HIF-1α, vimentin, NF-κB). Moreover, the presence of TAM cells is correlated with metastasis and inflammation by the secretion of different chemokines (EGF, TGF-β1, IL-6). In addition, pterostilbene down-regulated the NF-κB signaling pathway by the increased expression of miR-448 [181].

#### 4.3.3. Lignans

Lignans are plant secondary metabolites and some of them are considered phytoestrogens. They are found in high amounts in seeds (flaxseeds, sunflower) and nuts (peanuts, almonds, hazelnuts, pecan nuts, walnuts). Other food sources of lignans include cabbages (broccoli, cauliflower, kale), vegetables (avocado, eggplant, olives, tomatoes), gourds (pumpkin, zucchini), leaf vegetables (chicory, spinach, lettuce), celery stalks, fennel, fruits (black grapes, pomegranate, blackberries, etc.), extra virgin oil, and whole grain cereals. The most studied lignans are pinoresinol, lariciresinol, secoisolariciresinol, and matairesinol [182]. Several published papers have demonstrated the positive relationship between lignans intake and decreased incidence of breast, colorectal, and prostate cancer [182,183,184] through (i) the down-regulation of NF-κB/ HIF1-α signaling pathways, (ii) the decreased activity of the PI3/AKT signaling pathway, (iii) the inhibition of VEGF activity, and (iv) increased apoptosis through Bcl-2 down-regulation. The health-promoting effects of lignans are due to their main gut metabolites (entrolactone, enterodiol) [185]. Recent integrative and molecular docking research revealed the important role of enterolactone on breast cancer stem cells through their interaction with several targets (EGFR, Akt1, SMAD 2, SMAD 3, MMP-2, MAPK 8, EZH 2—a histone methyl transferase), the down-regulation of Wnt/β-catenin/PI3/Akt/mTOR signaling pathways, and epigenetic modifications (de-regulated expression of miR-30b, miR-324-5p, miR-382, and miR-423-3p and increased expression of miR-30b and miR-324-5p) [186,187].

#### 4.3.4. Other Non-Flavonoid Compounds

Oleacein, oleocanthal, hydroxytyrosol, oleuropein. Extravirgin olive oil is a key component of the Mediterranean diet. It consists of 98% triglycerides, made up of monounsaturated fatty acids (oleic acid), polyunsaturated fatty acids (linolenic acid, linoleic acid), and saturated fatty acids (palmitic acid, stearic acid). The remaining 2% is composed of natural antioxidants (hydroxytyrosol, oleuropein, oleacein, oleocanthal, decarboxymethyl oleuropein aglycone) [188]. The phenolic fraction of virgin olive oil (rich in simple phenols—tyrosol, hydroxytyrosol, hydroxytyrosol acetate—and phenolic secoiridoids—oleuropein, oleacein) showed promising effects in CSC through (i) the inhibition of epithelial to mesenchymal transition (EMT), mediated by TGF-β, (ii) the prevention of SMAD 4 and SNAIL 2 up-regulation, (iii) the down-regulation of vimentin and fibronectin, and (iv) suppressed Warburg effects [189,190]. According to recent research, microparticles containing oleuropein have shown anti-tumor effects on MCF-7 breast CSC population by suppressing EMT, down-regulating vimentin and Slug proteins, and decreasing proliferation by triggering p21/survivin expression [191].

Hydroxytyrosol. This compound extracted from olive oil was shown to inhibit CD44^+^/CD24^−^ stem cells derived from triple negative breast cancer cells (SUM159PT, BT549, MDA-MB-231, and Hs578T) by (i) suppressing Wnt/β-catenin signaling pathways, (ii) decreasing p-LRP6, LRP6, β-catenin, and cyclin D1 protein expression, (iii) down-regulating EMT markers (SLUG, ZEB1, SNAIL, and vimentin), and (iv) down-regulating TGF-β [192]. In addition, hydroxytyrosol inhibited the growth of prostate cancer stem cells through the down-regulation of MAPK, Akt, JAK/STAT, NF-κB, and TGF-β [193].

Curcumin. Curcumin is found in high concentrations in the Indian spice (*Curcuma longa* L.) but is also used in the Mediterranean diet along with other spices (ginger, rosemary, or black cumin) [111]. One of the mechanisms involved in curcumin anticancer properties is linked to cancer stem cells [194,195,196]. Curcumin showed promising results on colorectal cancer stem cells by the down-regulation of irinotecan/5-FU chemoresistance, decreased tumor sphere formation, the increased activity of Bax proteins, caspase-3, -8, -9 and decreased activity of the Bcl-2 protein [197,198]. In addition, curcumin micelle formulation targets colorectal cancer stem cells and decreases chemoresistance to oxaliplatin [199]. Curcumin reduced the expression of stem cell markers (DCLK1/CD44/ALDHA1/Lgr5/Nanog) in HCT-116, DLD-1, and HT-29 colon cancer cells, in both in vitro and in vivo studies [200]. Recent research has shown that curcumin targets cancer stem cell phenotypes in ex vivo models of colorectal liver metastases, through increased apoptosis and decreased levels of cancer stem cell markers (Nanog, Oct-3/4, HNF/FoxA2, VEGFR, ALDH1) [201]. Curcumin presented positive outcomes on breast cancer stem cells by the inhibition of microtentacles that persist in mammospheres and promote reattachment. Microtentacles are tubulin-based protrusions of the plasma membrane, which form in response to extracellular matrix detachment, that further promote metastasis [202]. In addition, curcumin down-regulates the expression of cancer stem cell markers (CD44 in MDA-MDB-231 cell line) and Hedgehog signaling pathways (in SUM159 and MCF7 cell lines). CD44 is a cell surface receptor involved in metastasis, cell proliferation, and angiogenesis [203,204]. According to Chen et al. (2017), curcumin have positive effects upon triple negative breast cancer mouse models through the inhibition of tumor growth, significantly reduced levels of CD44^+^/CD133^−^ stem cells, the inhibition of β-catenin and androgen receptor in nuclei, and the decreased expression of ALDH1 [205,206]. Treatment with curcumin significantly decreased Slug and CD24 protein expression levels in MDA-MDB-231 cell lined, along with the down-regulation of EMT and increased miR-34 activity [207]. Furthermore, curcumin inhibited EMT transition in MCF-7 breast cancer stem cells treated with endoxifen and TGF-β [208]. In addition, curcumin inhibited HIF-1α and HIF-2α expression in breast cancer stem cells [209]. According to Yang K et al. (2020), curcumin combined with glucose nanogold particles (Glu-GNPs) reduced breast cancer stem cell resistance to radiotherapy/chemotherapy through the reduced expression of HIF-1α and HSP90 (heat shock protein) and the down-regulation of ABCB1/ABCG2 efflux transporters [210,211]. Regarding curcumin effects in prostate CSCs, recent research has shown that curcumin significantly decreased CD133^+^ DU-145 cells and inhibited the spheres formation with a significant decrease in tumor size (preclinical research) [212]. In addition, curcumin increased the expression of tumor suppressors microARNs (miR-383-5p, miR-708) [213].

Gingerols and shagaols. Gingerols and shagaols are important phenolic compounds found in ginger roots (*Zingiberis officinalis* L.). An aqueous ginger extract showed positive effects on MDA-MDB-231 breast CSC (CD44^+^/CD24^−^ populations) by (i) the down-regulation of Oct3/4, Sox2 and Nanog, (ii) the increased expression of miR-200c, miR-30a, and miR-128a and (iii) the down-regulation of DNA methylation and drug resistance [214]. 6-gingerol inhibited tumor sphere formation in breast cancer, through (i) the down-regulation of Nanog, Oct-3/4, Sox-2, (ii) the up-regulation of p53 and Bax proteins, (iii) the down-regulation of Bcl-2 anti-apoptotic proteins, and (iv) the inhibition of STAT3 [215].

### 4.4. Non-Phenolic Compounds

#### 4.4.1. Carotenoids

Carotenoids are well-known antioxidants found in vegetables (carrots, tomatoes, endive, cichory leaves, dry beans, lentils, pumpkin, spinach), fruits (avocado, watermelon, kaki, seabuckthorn, *Citrus* species), spices (saffron), and marine organisms (seaweeds, shrimps) [216,217]. It is well known that carotenoids (beta carotene, lycopene) are the main sources of vitamin A for humans. All trans retinoic acid, a vitamin A liver metabolite, has shown a positive effect upon breast cancer stem cells by decreasing their capacity for self-renewal and enhancing their sensibility to doxorubicine [218].

Lycopene. In a mouse model of prostate cancer, a tomato diet (rich in lycopene) down-regulated the expression of stem cell-related genes, namely aldehyde dehydrogenase 1A1 gene (*Aldh1a1*) [219]. To the best of our knowledge, direct evidence that lycopene may act on stemness markers, such as Notch, Wnt/β-catenin, or Sonic Hedgehog pathways in prostate cancer has not yet been reported. Recent reports indicated that lycopene, in combination with enzalutamide, a competitor for androgen binding to its receptor, reduced the proliferation and invasion of castration-resistant prostate cancer cell lines and bone metastasis in animal models [220]. Additional experiments regarding the effect of lycopene or combination of lycopene and enzalutamide on prostate cancer stemness markers might be of therapeutic interest.

β-carotene. According to recent research, β-carotene decreased the number of colonospheres in CD133^+^/CD44^+^ colorectal cancer cells through (i) the up-regulation of histone H3/4 acetylation, (ii) the down-regulation of DNA methylation, and (iii) decreased oncogenic miRNA-1260b and miRNA-296-3p [221]. Moreover, beta carotene inhibited stemness markers (ALDH1, Notch, Sox2, and β-catenin) in CD133^+^CD44^+^ HT-29/HCT-116 cell lines [222]. A combination of beta carotene and oxaliplatin suppressed the colony number and down-regulated stemness markers (CD133, Sox2, and Oct4) in HCT-116 cell lines along with the inhibition of the JAK/STAT signaling pathway [223]. Combinations between β-carotene and 5-fluorouracil (5-FU) administrated through nanoparticles coated with hyaluronic acid have been recently reported in CRC cells resistant to 5-FU with promising results. This may represent a new strategy to overcome chemoresistance in colorectal cancer cells, through the down-regulation of ABC transporters genes [224]. Nevertheless, the outcomes of combinations between natural products and chemotherapeutic drugs on key markers of stemness, such as transcription factors (Nanog, Sox2, Oct3/4) or signaling pathways (Notch, Wnt/β-catenin, Sonic Hedgehog) in CRC remain to be elucidated.

Lutein. Hypoxia promotes metastasis, epithelial to mesenchymal transition, the activation of Notch signaling pathways, and increases the expression of Notch ligands (JAG1, JAG 2), the activation of HIF-1α, and the activation of transcription factors involved in Notch signaling (HES-1). Treatment with lutein of MCF-7 breast cancer cells cultured under hypoxic conditions decreased EMT, Notch-3, and HES-1 [225].

Astaxanthin. This carotenoid is mainly found in algae and marine organisms (shrimps). According to recent research, it has a positive effect on breast cancer stem cells through (i) the reduction of colony and spheroid formation, (ii) the down-regulation of Oct4, Nanog, and mutant p53 expression, (iii) decreased invasion and metastasis [226].

Fucoxanthin. A marine carotenoid found in numerous classes of microalgae and macroalgae (brown ones) is fucoxanthin. This compound showed promising effects in breast cancer stem cells through decreased mammosphere formation [227].

Saffron. A saffron extract had positive effects on colorectal cancer stem cells by down-regulating metastasis associated with colon cancer 1 (MACC1). MACC1 is also linked to epithelial to mesenchymal transition and is considered a poor prognosis marker in colorectal cancer. Saffron extract decreased the expression of DCLK1 (double cortin like kinase 1), which is a putative cancer stem cell marker associated with colorectal cancer [228]. Crocin showed positive results on MDA-MDB-231 triple negative breast cancer cells, through the down-regulation of EMT [229]. According to recent research, a saffron extract (with 5 mM crocin, 5 μM crocetin, 5 mM safranal) showed promising results on prostate cancer stem cells through (i) increased apoptosis (increased activity of Bcl-2 protein), (ii) the down-regulation of histone lysine methyltransferase 2 (EHMT2), as well as NAD-dependent protein deacetylase sirtuin 1 (SIRT1), (iii) the up-regulation of p53, (iv) the down-regulation of DNMT3b, and (v) decreased levels of CD44, NF-κB, TNF-α, c-Myc [230].

#### 4.4.2. Triterpenic Compounds

Among triterpenic compounds, characteristic to a Mediterranean diet, ursolic acid and oleanolic acid are found in spices (thyme, sage, and basil) [111].

Ursolic acid. This acid showed promising results on breast cancer stem cells (in vitro and in vivo) by (i) decreasing mammosphere formation, (ii) down-regulating ALDH1, (iii) down-regulating stemness markers (Sox2, Oct4, c-Myc), (iv) up-regulating ferroptosis, (v) increased levels of lipid peroxidation and ROS accumulation, and (vi) the decreased expression of Nrf2 [231]. In addition, ursolic acid inhibited colony formation in MCF-7 mammospheres and down-regulated ERK, PI3K/AKT signaling pathways [232]. According to Liao et al. (2023), ursolic acid showed inhibitory effects upon the stemness of MDA-MDB-231 and MCF-7 breast cancer cells by means of (i) the down-regulation of Nanog and Oct4 genes, (ii) decreased EMT, (iii) the reduction of oncogenic micro RNA (miR-9 and miR-221), (iv) the inactivation of FAK/PI3K/Akt/mTOR signaling pathways, and (v) the decreased activity of argonaute-2 (AGO2), a key regulator of microRNA biogenesis [233].

Oleanolic acid. This acid had positive results upon colorectal cancer stem cells through the down-regulation of the JAK2/STAT3 signaling pathway, improved response to 5-fluorouracil treatment, and the decreased viability of cancer cells [234].

#### 4.4.3. Vitamin E (Tocopherols and Tocotrienols)

Tocotrienols and tocopherols are found in high amounts in pecan nuts, pine nuts, almonds, hazelnuts, pistachio, and cashew [235]. According to recent research, tocotrienols have anti-cancer (for breast, colorectal, and pancreatic tumors) effects through various mechanisms: (i) decreased tumor growth and the increased expression of pro-apoptotic proteins, (ii) the increased expression of p21 and p27, (iii) the down-regulation of AKT and NF-κB activity, and (iv) the down-regulation of VEGF, cyclin D1, c-Myc, MMP-2, and COX-2 [236].

γ-tocotrienol. This tocotrienol inhibits mammosphere formation in breast cancer stem cells, through the down-regulation of the Ras/ERK pathway and Src homology 2 domain-containing phosphatase 1 (SHP1) and 2 (SHP2) genes. Still, γ-tocotrienol did not affect the self-renewal capacity of CSCs, with no influence on TGF-β [237]. Furthermore, γ-tocotrienol inhibits Wnt-β catenin signaling pathways and epithelial to mesenchymal transition in breast cancer stem cells [238]. γ-tocotrienol is also an effective agent in targeting prostate cancer stem cells through the down-regulation of stemness markers (CD133, CD44), the elimination of chemoresistance, and decreased viability (both in vitro and in vivo) [239]. Another isomer, δ-tocotrienol, showed promising results on prostate cancer stem cells through the down-regulation of HIF-1α and HIF-2α [240].

#### 4.4.4. Nitrogen Compounds

Capsaicin. Chili pepper-derived compounds lower the risk of colorectal cancer, due to an increase in butyrogenic bacteria and the up-regulation of *Firmicutes/Bacterioides* ratio and *Faecalibacterium* abundance [241]. According to recent research, short-chain fatty acids (mainly butyrate) might influence colorectal cancer stem cells through the down-regulation of Wnt and PI3K/AKT/mTOR signaling pathways and the up-regulation of secreted Frizzled-related protein expression in the Hedgehog signaling pathway (a natural inhibitor of Wnt), the up-regulation of p21 expression, or the inhibition of Notch signaling [242]. Capsaicin has an anti-tumor effect on breast cancer stem cells through the down-regulation of the Notch signaling pathway and decreased mammosphere formation of CD44^+^/CD 24^−^ cancer cells [243]. Furthermore, capsaicin exerts an inhibitory effect on prostate cancer stem cells through the suppression of the Wnt/β-catenin signaling pathway and the down-regulation of GSK-3, cyclin D1, and c-Myc [244].

Piperine. The main compound in black pepper, piperine, plays a key role in increasing curcumin bioavailability, but it is also used for its anti-cancer properties. Piperine enhances doxorubicine sensitivity in triple negative breast cancer cell lines by down-regulating PI3K/AKT/mTOR signaling pathways and suppressed ALDH1 expression [245]. In addition, a standardized extract from *Piperum longum* L. with 25.04% piperine, 2.91% pipernanoline, and 0.61% guinesine showed promising results on CD44^+^/CD24^−^ breast cancer stem cells through decreased mammosphere formation and the down-regulation of stemness markers (Nanog, Oct4, Sox2, and EpCAM) [246].

#### 4.4.5. Organosulfur Compounds

*Allium sativum* L. (garlic) is a rich source of organosulfur compounds, mainly allicin, diallyl disulphide, and diallyl trisulphide. Several mechanisms are involved in its antitumor effect: (i) the inhibition of angiogenesis, (ii) the inhibition of tumor inflammation, (iii) the inhibition of metastasis, (iv) genomic instability, (v) replicative immortality, (vi) anti-growth signal evasion, (vii) apoptosis induction, and (viii) tumor metabolism dysregulation [247]. Moreover, garlic organosulphur compounds and garlic polysaccharides interact with gut microbiota, providing chemopreventive effects on colorectal cancer. According to recent research, garlic polysaccharides have an anti-inflammatory effect (by decreasing IL-1β, IL-6, and TNF-α) and increase the production of short-chain fatty acids, whilst organosulfur compounds increase the *Firmicutes/Bacterioides* ratio and decrease *Actinobacteria* [241]. Regarding the role of garlic and its main constituents on cancer stem cells, a hydroalcoholic garlic extract (obtained using fresh cloves treated with 40% ethanol), which contains diallyl sulfide, diallyl disulfide, dipropenyl disulfide, and allyl methyl trisulfide (identified using GC-MS analysis), reduced the growth of CD133+ MCF-7 breast cancer stem cells and inhibited the epithelial to mesenchymal transition, induced by hypoxia [248].

Diallyl trisulfide. According to recent research, diallyl trisulfide has shown anticancer effects in breast cancer stem cells by decreasing ALDH activity and down-regulating forkhead box Q1 (FOXQ1) protein activity. It is well known that the overexpression of FOXQ1 is linked with stemness and mammosphere formation [249]. Moreover, diallyl trisulfide (DATS) suppresses the activity of breast cancer stem cells by the down-regulation of stemness markers (Nanog, Oct4, ALDH1, CD44), increased apoptosis (up-regulation of Bax protein, caspase-8, caspase-9, caspase-3), and the inhibition of Wnt/β-catenin signaling pathways [250]. In vitro and in vivo studies using diallyl disulfide showed promising results on breast cancer stem cells by the inhibition of tumor growth and metastasis; the down-regulation of PMK2/AMPK; and decreased glucose uptake and lactate production [251].

Phenethyl isothiocyanate. Important glucosinolates are also found in *Brasicaceae* sp. (broccoli, kale, cabbage, cauliflower, watercress, etc.). Among them, sulforaphane, phenethyl isothiocyanate, and benzyl isothiocyanate are intensively studied for their antitumor effects and they act through (i) epigenetic modulation, (ii) the induction of cell cycle arrest, (iii) the activation of apoptosis, (iv) the inhibition of proliferation and metastasis, (v) the inhibition of histone deacetylase, (vi) the regulation of fatty acid synthesis, and (vii) the induction of antioxidant pathways [252,253]. Phenethyl isothiocyanate showed promising results on colorectal cancer stem cells (in vitro and in vivo) by decreasing the expression of stemness markers (Nanog, Oct4, Sox2, ALDH1, CD44, EpCAM), inhibiting EMT, and down-regulating ERK and phosphor-Smad2/3. The pre-treatment of mice with induced colorectal cancer, with phenethyl isothiocyanate, led to a decrease in tumor growth, through the down-regulation of several genes (CXCL2, CXCL3, IL17C, IL1A, TNFAIP2, etc.) associated with inflammation, cytokine activity, or immune response [254]. In addition, phenethyl isothiocyanate inhibited breast cancer stem cells through the epigenetic reactivation of CDH1 (cadherin 1). The CDH1 gene encodes a glycoprotein, E-cadherin, which acts as a tumor suppressor. CDH1 expression is usually silenced in solid tumors due to DNA hypermetylation [255]. Research regarding watercress (rich in phenethyl isothiocyanate) and broccoli (containing sulforaphane, 3-butenyl isothiocyanate) extracts have revealed positive outcomes on colorectal cancer stem cells, through decreased viability, decreased ALDH1 activity, increased cyclin A2 levels (which negatively influence cell motility and metastasis), and the down-regulation of CDH1, vimentin, and Wnt/β-catenin [256].

Sulforaphane. The main organosulfur compound found in broccoli, sulforaphane, inhibits breast cancer stem cells (in vivo and in vitro) through decreased mammosphere formation, decreased ALDH1 population, and the down-regulation of Wnt-β catenin [257]. Moreover, a formulation containing sulforaphane and cyclodextrin reduced breast cancer stem cell viability in primary and metastatic ER+ samples from patients, through decreased ALDH positive population, the prevention of tamoxifen enrichment and the inhibition of metastasis and STAT3 activity [258]. Chemotherapeutic agents (like docetaxel or paclitaxel) induce Il-6/IL-8 secretion with an expansion of cancer stem cells in triple negative breast cancer. However, the association of docetaxel with sulforaphane (in vitro and in vivo) conducts to a greater reduction in tumor growth and inhibits the generation of secondary tumor formation related to the primary treatment. Moreover, sulforaphane reduces stem markers (EpCAM, ALDH1, CD44^+^, CD24^−^) and down-regulates NF-κB and p65 [259]. According to Vyas et al. (2016), sulforaphane inhibits prostate cancer stem cells by the down-regulation of the oncogenic c-Myc protein, without affecting basal glycolysis (which is promoted by c-Myc overexpression) [260]. In addition, a combination of human tumor necrosis factor-related apoptosis ligand (TRAIL) and sulforaphane is superior compared with single treatments in reducing prostate cancer stem cells and self-renewal characteristics. Furthermore, the combination significantly down-regulated Oct-3/4, VEGFR2, Snail, Otx2, Sox2, Nanog, and ALDH1, and inhibited EMT in vitro. In vivo TRAIL and sulforaphane combinations reduced tumor growth and cancer stem cells markers [261].

#### 4.4.6. Omega-3/Omega-6/Omega-9 Fatty Acids

Omega-3 fatty acids, namely α-linolenic acid, eicosapentaenoic acid (EPA), and docosahaexenoic acid (DHA) are polyunsaturated fatty acids (PUFAS) found in fish (herring, salmon, mackerel), marine organisms, vegetable oils (linseeds, rapeseeds, blackcurrant seeds), and nuts [262]. Walnuts are an excellent source of α-linolenic acid (9 g/100 g) [263]. Nuts contain high amounts of omega-6 fatty acids (linoleic acid). Almonds, pistachio, and walnuts are among the richest sources (walnut—38 g/100 g linoleic acid, pistachio—14 g/100 g, and almond—12.32 g/100 g) [263].

Omega-3 fatty acids. EPA showed promising results on colorectal stem cells by decreasing stemness marker (CD133) and increasing the colonic epithelium differentiation markers (mucin 2 and cytokeratin 20) [264]. It also increased sensitivity to 5-FU or mitomycin treatment [264,265]. According to Sam R et al. (2016), DHA is more potent than EPA, or their combination, on colorectal cancer stem cells in terms of decreased viability, the inhibition of growth, and the induction of apoptosis. Still, their combination was more potent in inducing survivin down-regulation than either treatment with EPA or DHA alone [266]. Moreover, DHA increased miR-161-1 expression and up-regulated p-53 in colorectal cancer stem cells [267]. Volpato M et al. (2020) demonstrated in a preclinical study that the inhibition of COX activity by aspirin or celecoxib increased colorectal cancer stem cells sensitivity to EPA, via a reduction in EPA catabolism and the increase in its intracellular level [268].

Oleic acid. Cancer stem cells rely on increased lipogenesis for self-renewal and growth. Several enzymes, such as fatty acids synthase (FAS) and fatty acid desaturases (FADS1, FADS2), and stearoyl-CoA desaturase-1 (SCD1), are responsible for maintaining cancer stemness. Cancer stem cells require more monounsaturated fatty acids (MUFAs), thus lipid desaturation is considered as a hallmark of cancer stem cells; in addition, SCD1 up-regulates Wnt/β-catenin signaling pathways, a key factor involved in cancer stem cell proliferation [269]. Oleic acid, an important omega-9 fatty acid, is the main compound found in extravirgin/virgin olive oil. According to recent research, oleic acid inhibits the growth of breast cancer stem cells (MDA-MDB-231, MCF-7 cell lines); it decreases MRP1 and β-catenin levels, suppresses the invasion and metastasis, through the induction of ROS (reactive oxygen species), and down-regulates FAK/AKT/NF-κB signaling pathways [270].

On the other hand, oleic acid showed opposite effects on different cancer cell lines, as follows: oleic acid inhibited the growth of MCF-7, SUM225, HCC1354, and MDA-MDB-231 but significantly increased the proliferation of MCF10DCIS (a ductal carcinoma in situ cell line), through lipid loading and the up-regulation of lipogenic genes (SREBP-1, FAS, ACC1). Oleic acid also promoted the proliferation of the CD44^+^/CD24^−^/ALDH1^high^ population [271]. Other authors found that oleic acid promotes cancer stemness by the up-regulation of stearoyl-CoA-desaturase (SCD), which is associated with disease progression in colorectal cancer patients [272,273].

#### 4.4.7. Aromatic Compounds

Eugenol. Clove (*Syzygium aromaticum* (L.) Merr. and L.M. Perry) buds are an important source of eugenol. Recent research has highlighted their importance as a chemopreventive agent in different types of cancer [241]. According to Choudhury et al. (2020), eugenol restricts cancer stem populations in breast cancer through the down-regulation of β-catenin and the restriction of colony formation and stemness, along with the down-regulation of mRNA expression in several markers (Oct4, Notch1, EpCAM) [274]. Moreover, eugenol enhanced cisplatin activity in triple negative breast cancer stem cells (in vitro and in vivo) by means of ALDH enzyme inhibition, the down-regulation of NF-κB signaling pathways, the inhibition of EMT, the down-regulation of MMP-2/MMP-9, and the inhibition of angiogenesis [275].

Cinnamaldehyde. The main constituent of cinnamon essential oil, cinnamaldehyde, showed anticancer effects in colorectal cancer stem cells through increased sensitivity to oxaliplatin treatment, reversed hypoxia-induced epithelial to mesenchymal transition, and the down-regulation of Wnt/β-catenin signaling pathways [276]. Another compound, 2-hydroxycinnamaldehyde, showed promising results on triple negative breast cancer cell lines through (i) the inhibition of epithelial to mesenchymal transition, (ii) the down-regulation of vimentin, Snail transcriptional factor, and EGF, (iii) the up-regulation of GSK-3β nuclear level, and iv) the down-regulation of transcriptional factors Id-1 and SP1, involved in breast cancer invasion [277].

Thymoquinone. This is the main compound of *Nigella sativa* L. (black cumin) essential oil, which is intensively studied for its antitumor properties, due to its safe profile. According to recent research, thymoquinone exerts anti-neoplastic effects in a wide range of cancers including colorectal, breast, and prostate tumors, alone or in combination with chemotherapeutic agents (cisplatin, cabazitaxel, docetaxel, cyclophosphamide, etc.) or other natural compounds (genistein, ferulic acid, emodin, resveratrol, etc.). The molecular mechanisms involved in thymoquinone anticancer effects include (i) cell cycle arrest, (ii) the down-regulation of cyclin D1 and JAK2/STAT3, PI3/Akt/mTOR, NF-κB signaling pathways, (iii) the activation of p53, (iv) increased Bax/Bcl-2 ratio and caspases activity, (v) the down-regulation of beclin 1 and MMP-2/MMP-9, (vi) the decreased expression of survivin, (vi) the increased release of mitochondrial cytochrome-c, (vii) the down-regulation of Notch and Wnt/β-catenin, (vii) the inhibition of angiogenesis (down-regulation of EGF, VEGF) [278], and viii) the inhibition of glycolytic metabolism (Warburg effect), through the down-regulation of hexokinase 2 [279]. Thymoquinone effects on cancer stem cells, Bashmail et al. (2020), showed that thymoquinone alone or in combination with paclitaxel decreased CD44^+^/CD24^−^ stem cell clones in both MCF-7 and T47D cells (breast cancer) through the inhibition of EMT and the down-regulation of TWIST-1 gene [280]. Moreover, a combination of docetaxel and thymoquinone in nanoemulsion exhibited a significant decrease in breast cancer stem cells through a decreased expression of Snail 1 and Twist 1 transcriptional factors [281]. In addition, thymoquinone down-regulated the expression of Wnt/β-catenin and vasculogenic factors in breast cancer stem cells [282]. Furthermore, thymoquinone induced apoptosis in 5-FU resistant colorectal stem cells (in vitro and in vivo) through the inhibition of self-renewal capacity, reduced invasion, and the inhibition of colonospheres formation (by p21 up-regulation and down-regulation of NF-κB, p-MEK, and PCNA). Thymoquinone also showed DNA damage by increasing H2AX (eukaryotic histone). In addition, it caused a significant reduction in EpCAM expression and proliferation marker Ki67 and up-regulated cytokeratin epithelial markers (CK-8, CK-19) [283].

#### 4.4.8. Dietary Fibers

Inulin. A natural polysaccharide, inulin, is commonly known as a prebiotic agent. It is found in leek, onion, garlic, banana, fresh *Asparagus officinalis* stems, wheat, and rye. Inulin, along with other fructooligosaccharides (FOS), promotes the growth of health beneficial groups of colonic microorganisms (*Lactobacillus acidophilus*, *L. casei*, *L. delbruekii*; *Bifidobacterium bifidum*, *B. longum*, *B. infantis*, *B. adolescentis*; Streptococci—*Streptococcus salivarius*, *S. lactis*) and down-regulates *Proteobacteria, Desulfovibrio*, *Lactococcus* [284,285]. The healthy bacteria promote the production of short-chain fatty acids, with anti-inflammatory effects and the prevention of carcinogenesis [284]. Moreover, lactic acid along with short-chain fatty acids (SCFA) lowers the pH, thus inhibiting the growth of enteropathogenic bacteria. In addition, *Lactobacillus* sp. and *Bifidobacteria* sp. produce peptides (lantibiotics, bactriocins, bacteriolysins, reuterin) that directly inhibit the growth of pathogenic bacteria [284]. The link between dysbiosis and colorectal cancer consists in several metabolites (polyamines, hydrogen sulfide, colibactin, indole derivatives, trimethylamine N-oxide, etc.) formed by pathogenic bacteria (*Escherichia coli*, *Fusobacterium nucleatum*, *Desulfovibrios G*, *Clostridium*, *Enterococcus faecalis*) [286]. According to recent research, SCFA such as propionate, acetate, and butyrate block Notch signaling, down-regulate PI3K/AKT/m-TOR, Hedgehog, Hippo, and NF-κB signaling pathways and block HIF-1α stimulation, which are essential molecular mechanisms involved in colorectal cancer stem cell proliferation. Moreover, butyrate inhibits STAT3 signaling, thereby down-regulating the expression of c-Myc, Bcl-2, cyclin D1, and HIF-1α. Butyrate is considered a pro-ferroptotic agent, thus suppressing CD44 and xCT expression in colorectal cancer stem cells. In addition, butyrate was found to boost the pro-ferroptotic effect of oxaliplatin (OXA) [287]. According to recent research, acetate promotes colon cancer cells apoptosis by increased caspase-3 activity, whilst propionate down-regulates the expression of arginine methyltransferase, increases p21 and p53 expression, and decreases survivin levels [242]. SCFA favor the expression of tissue inhibitor matrix metalloproteinases (TIMPS), which attenuate metastasis and inhibit histone deacetylases [242,285]. Moreover, SCFA maintain immune homeostasis [285].

## 5. Major Mechanisms of Action in Case of Dietary Compounds in CRC, BC, and PCa Cancer Stem Cells

### 5.1. Signaling Pathways

**Figure 1:**
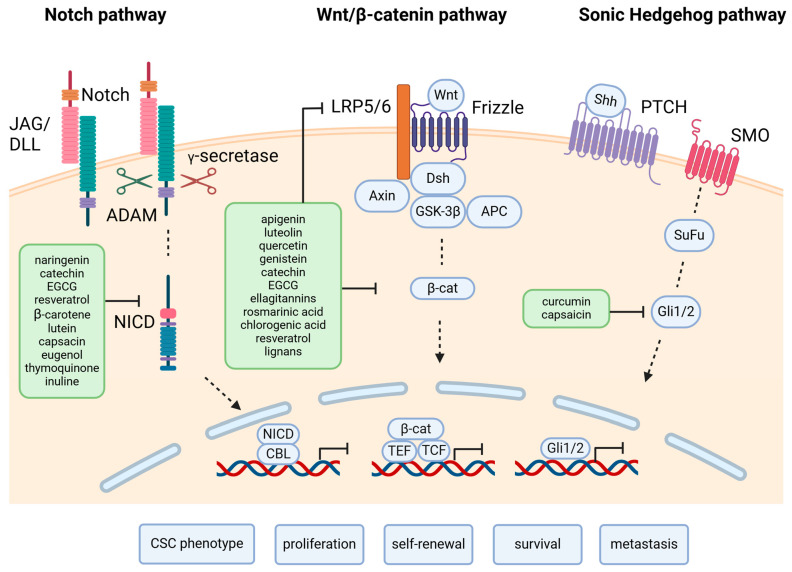
Schematic representation of major mechanisms of action in cases of phenolic and non-phenolic dietary compounds on stemness pathways, such as Notch, Wnt/β-catenin, and Sonic Hedgehog pathways; insights into breast, prostate, and colorectal cancer. In active CSC, (i) after heterodimerization of Notch with JAG/DLL and proteolysis with ADAM and γ-secretase, a soluble fragment NICS is liberated in cytoplasm and translocated into nucleus; (ii) Wnt binds to Frizzled and its co-receptor LRP5/6 stimulating the complex formation (axin, Dsh, GSK-3β, APC) that will lead to β-catenin translocation into nucleus; (iii) Shh binds to PTCH which activate SMO and Gli1/2 is translocated into nucleus [290,293]. The phenolic and non-phenolic compounds can hinder the activity of these pathways and, eventually, will block gene transcription and further the processes associated with cell stemness, such as self-renewal and proliferation. Legend: JAG, Jagged ligand; DLL, Delta-like ligand; ADAM, a disintegrin and metalloproteinase; NICD, Notch intracellular domain; CBL, Casitas B-lineage Lymphoma transcription factor; Dsh, dishevelled; APC, adenomatous polyposis coli; GSK-3β, glycogen synthase kinase-3; LRP5/6, low-density lipoprotein receptor-related protein-5/-6; TEF, thyrotrophic embryonic factor; TCF, T-cell factor; PTCH, patched; Shh, sonic hedgehog; SMO, smoothened; SuFu, suppressor of fused; Gli1/2, glioma-associated oncoprotein family. Created with BioRender.com (accessed on 23 December 2024).

**Figure 2:**
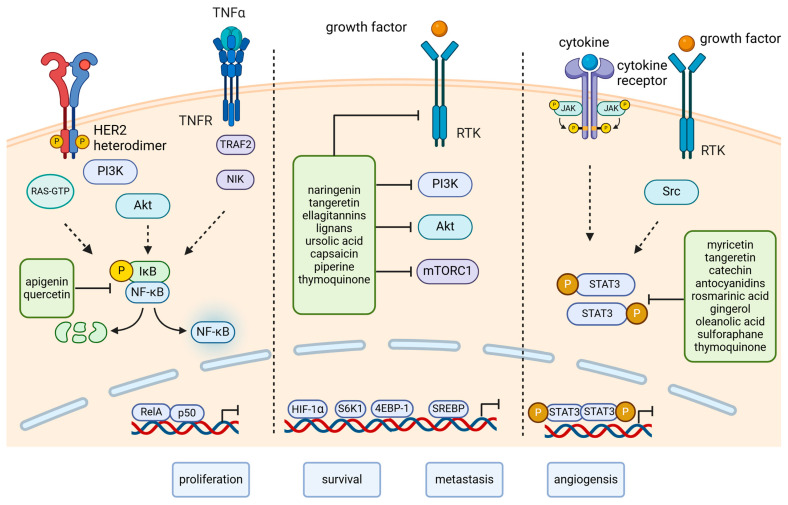
Schematic representation of major mechanisms of action in case of phenolic and non-phenolic dietary compounds on stemness markers such as NF-κB level, PI3K/Akt/mTOR, and STAT3 pathways; insights into breast, prostate, and colorectal cancer [290,294,298,300]. Legend: HER2, human epidermal growth factor receptor 2; Ras, protein similar to the one coded by Rat sarcoma virus; TNFα, tumor necrosis factor α, TNFR1, tumor necrosis factor receptor; TRAF, TNF Receptor-Associated Factor 2; NIK, NF-κB-inducing kinase; IκB, inhibitor of nuclear factor kappa B; NF-κB, nuclear factor kappa-light-chain-enhancer of activated B cells; RelA, member of NF-κB transcription factors (p65); RTK, receptor tyrosine kinase; PI3K, phosphoinositide 3-kinase; Akt, protein kinase B; mTORC1, mammalian target of rapamycin complex 1; HIF-1α, hypoxia-inducible factor 1α; S6K1, S6 kinase 1; 4EBP-1, eukaryotic translation initiation factor 4E-binding protein 1; SREBP, sterol regulatory element-binding protein; IL, interleukin; ILR, interleukin receptor; JAK, Janus kinase; Src, protein similar to the one coded by Rous sarcoma virus, non-receptor tyrosine kinase; STAT3, signal transducer and activator of transcription 3. Created with BioRender.com (accessed on 23 December 2024).

Complex signaling pathways are responsible in maintaining aberrant CSC activity that will conduct self-renewal, proliferation, heterogeneity, chemoresistance, and metastasis [288,289,290]. Among these intricate lines of signaling, this paper focuses on Notch, Wnt/β-catenin, and Hedgehog pathways (Figure 1), and NF-κB, growth factors, and STAT3 pathways (Figure 2) in CRC, breast, and prostate CSC. Notch pathways in CSC are responsible for self-renewal, differentiation, cell maintenance, and proliferation [290,291]. Wnt/β-catenin signaling pathways in stem cells are required for embryonic development, while aberrant expression in CSC has been connected to metastasis [292]. Hedgehog pathways in stem cells are essential for homeostasis and self-renewal, while in CSC, they are implicated in maintaining aberrant cell proliferation [292]. Experimental data introduced in Chapter 4, mostly in vitro and in vivo, demonstrated that dietary compounds (phenolic and non-phenolic) reduced Notch [15,16,222], Wnt/β-catenin [238,244,256], and Sonic Hedgehog [147,203,204] signaling pathways in CRC, breast, and prostate CSC.

Nuclear factor kappa-light-chain-enhancer of activated B cells (NF-κB) are a family of five members (NF-κB1, NF-κB2, RelA, RelB, and c-Rel) responsible for the immune and inflammatory cellular processes [294]. In addition, in CSC, nuclear factor NF-κB mediate several cellular activities regarding proliferation, self-renewal, and metastasis [295]. The PI3K/Akt/mTOR signaling pathway has been reported to be involved in self-renewal and increase the clonogenic ability, tumor formation, and maintenance of CSC [296]. In immune cells, STAT3 is activated via a canonical pathway, namely, interleukin receptor-Janus kinase [297]. Nevertheless, the stemness maintenance and survival of CSC has been demonstrated to be based on the activation of STAT3 [298] and, particularly, IL6/JAK/STAT3 is activated in breast cancer stem cells [298]. Several phenolic and non-phenolic dietary compounds have demonstrated their ability to reduce NF-κB levels [122,175,193], PI3K/Akt/mTOR [139,142,299], and STAT3 [234,258] pathways in CRC, breast, and prostate CSC.

### 5.2. Epithelial to Mesenchymal Transition

**Figure 3:**
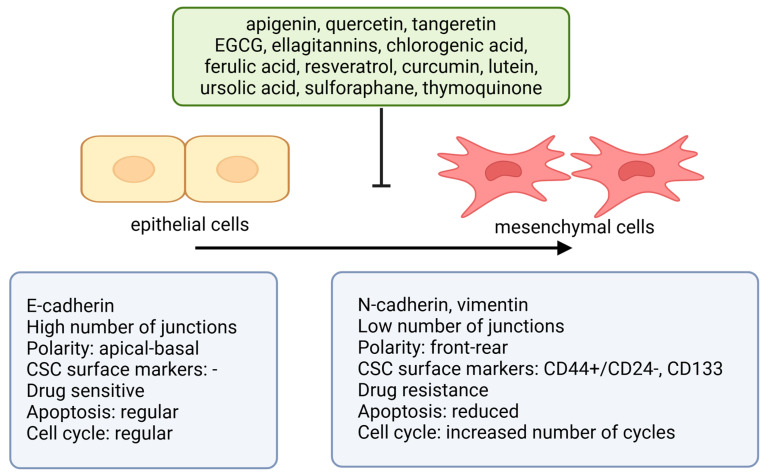
Phenolic and non-phenolic action on CSC that manifest mesenchymal phenotype in breast, prostate, and colorectal cancer [302,303]. Created with BioRender.com (accessed on 23 December 2024).

EMT and the inverse course of action, namely mesenchymal to epithelial transition (MET), are studied during embryonic development [301]. Normal EMT was observed in embryogenesis, organ development, wound healing, tissue regeneration with or without fibrosis. On the other side, in malignant transformation, EMT processes are associated with invasion and metastasis [301]. CSC acquire a series of features specific to EMT and they have the ability to migrate, to induce the modification of the extracellular matrix, and to find a proper niche to induce the formation of secondary tumors [302]. The ability of diet and functional food to partially reduce the malignant phenotype associated with EMT in CSC has been noticed in the case of CRC [154], breast [117,142], and prostate [130] CSC. A simplified graphic of the mechanism of action in cases of phenolic and non-phenolic dietary compounds on colorectal, breast, and prostate CSC presented in Chapter 4 is shown in Figure 3.

### 5.3. Other Mechanisms of Action

Additional mechanisms of action of the phenolic and non-phenolic diet in CRC, breast, and prostate CSC presented in Chapter 4 include reductions in surface markers, such as CD44 [124,138,218] and CD133 [16,138,144], reduction in transcription factors associated with stemness features, such as Sox2 [145,214,222], Nanog [214,215,226], and Oct3/4 [231,233,246], and chemoresistance markers, such as ALDH [245,249,250].

## 6. Cancer Cell Differentiation and Dietary Compounds

Only 1–4% of the cells from the tumor display stemness features [304] and these characteristics are associated with tumor heterogeneity, metastasis, and relapse [305]. However, most of the previously presented data are based mainly on the results from in vitro and in vivo experiments. As a result of missing published clinical data regarding the effects of dietary compounds on molecular mechanisms in CSC and with the aim of having a full picture of the up-to-date research in the field, this chapter briefly addresses the tumor grading or tumor differentiation (please, see “patient condition” from Table 4) in rapport with dietary compounds (Table 4). In the clinic, the diagnostic for solid tumors is based on histopathological data including tumor differentiation and on the evaluation of serum markers, such as carcinoembryonic antigen (CEA), prostate serum antigen (PSA), and other circulant markers [306,307,308,309]. Several markers are in use to establish the diagnostic or to monitor disease evolution; however, no specific serum marker was reported for BC [310]. Differentiation grades in solid tumors have been extensively reviewed elsewhere in case of CRC [311,312], BC [313,314], and PCa [315], with higher degrees of differentiation having better prognosis compared with lower ones [306]. In addition, in line with the proposed aim of this chapter, earlier data from the 1970s and further reports informed us about a possible therapeutic approach, particularly differential therapy, where CSC from acute promyelocytic leukemia (PML) are differentiated to a mature phenotype prone to senescence [316]. The administration of retinoic acid in combination with chemotherapy or arsenic trioxide induced PML remission in 95 and 100% of patients, respectively [317].

Cancer differentiation in solid tumors and dietary compounds is an extended topic that needs complex research investigation that cannot be covered in a single chapter belonging to a paper. Nevertheless, in this chapter, the data from the most examined dietary compounds in clinical studies are reported, such as curcumin, lycopene, PUFA, and vitamins in relationship with different stages of malignant pathology, from early stages with well differentiated tumors to advanced stages with low differentiated or undifferentiated tumors. Thus, liposomal curcumin reduced CEA levels in one patient with advanced CRC [318], while the administration of retinoic acid, a derivative of vitamin A in combination with low-dose interleukin 2, displayed improvements in progression-free survival and overall survival in metastatic CRC [334]. The addition of PUFA to Taxol therapy in patients with breast cancer (the tumor present and the stage not-specified) reduced peripheral neuropathy, a side effect of chemotherapy [325]. The administration of retinoic acid before paclitaxel in patients with advanced or recurrent breast cancer induced a 76% clinical benefit compared with paclitaxel alone [333]. In advanced metastatic prostate cancer, curcumin reduced PSA levels significantly [318], and in another study, co-treatment with docetaxel reduced the CEA level [319]. The administration of lycopene in localized prostate cancer decreased tumor size and PSA [320], while in metastatic castrate-resistant prostate cancer, the co-administration of docetaxel and lycopene induced a decrease in PSA levels and increased the median survival compared with docetaxel alone [321]. The administration of vitamin D3 in low-risk prostate cancer was correlated with a 55% decrease in Gleason score [331]. On the other hand, the supplementation of the treatment with isotretinoin, a derivative of vitamin A in metastatic prostate cancer did not modify PSA levels [334]. Taken together, part of the above-mentioned studies had shown beneficial results after administration to patients, particularly in the early stages of the disease or in combinatorial therapy. However, several drawbacks need to be mentioned, and these might include the following: the limited number of studies, the low number of patients included in the trials, the contradictory effects (both the increase and decrease of the markers), or no effects. Additional studies are required to clarify the clinical benefit of dietary compounds on cancer cell differentiation and, in addition, the focus on stemness markers might represent a future therapeutic approach.

## 7. Conclusions

This paper offered a large amount of information regarding the activity of well-known compounds found in food/beverages in CSC from pathologies with high incidence in human population such as CRC, BC, and PCa. These effects are usually seen at higher concentrations than those obtainable by food intake; however, there are data suggesting that the effects might be additive and that complex diets enriched in the compounds described in this review are indeed associated with lower cancer risks.

## Figures and Tables

**Table 1:** Markers for stem cells in colon, breast, and prostate tissue.

Marker	Function	Colon	Breast	Prostate
ALDH1	Enzyme for cellular detoxification	[26]	[27]	NA
Bcl-2	Anti-apoptotic protein	NA	NA	[22]
CD133	Marker for stem and progenitor cells	[28]	[29]	[22]
CD24	Cell adhesion and signal transduction	NA	[30]	NA
CD44	Cell–cell interactions, migration, and adhesion	NA	NA	[31]
CD49f	Integrin, cell adhesion, and signaling	NA	[32]	[31]
Hopx	Regulates stem cell quiescence	[33]	NA	NA
Lgr5	Part of Wnt signaling pathway	[33]	NA	NA
Lrig1	Regulates EGFR signaling	[33]	NA	NA
OLFM4	Cell adhesion, tumor growth	[34]	NA	NA
p27	Cell cycle inhibitor, regulates stem cell quiescence	NA	NA	[31]
Sca1	Stem cell lineage fate	NA	[35]	[31]

Legend: NA, not applicable.

**Table 2:** Markers for CSC in cases of colorectal cancer (CRC), breast cancer (BC), prostate cancer (PCa).

Marker	Function	CRC	BC	PCa
ALDH1	Enzyme for cellular detoxification	[26]	[66,67]	[72]
CD44	Cell–cell interactions, migration, and adhesion	[74]	[66,67]	[70]
CD49f	Integrin, cell adhesion, and signaling	[75]	[66]	[71]
CD133	Marker for stem and progenitor cells	[73]	[66,67]	[69]
EZH2	Histone methyltransferase, gene silencing	NA	NA	[72]
SOX2	Transcription factor, stem cell pluripotency	NA	[68]	[72]
Integrin α2β1	Cell adhesion, extracellular matrix binding	NA	NA	[70]

Legend: NA, not applicable.

**Table 3:** Dietary phenolic and non-phenolic compounds and their sources [97,102,103,104,105,106,107,108,109,110,111,112,113,114].

Dietary Class	Representative Compounds	Sources
PHENOLIC COMPOUNDS		
**Flavonoids**		
Flavones	apigenin, luteolin, scutellarein, isoscutellarein	celery, green pepper, onion, thyme, mountain tea (flowers, leaves and stems of *Sideritis* sp.), apples, grapes
Flavonols	quercetin, kaempferol, myricetin	broccoli, lettuce, kale, onion, apple, grapes, cappers, fennel (whole plant), parsley
Flavanones	naringenin, hesperitin, diosmetin, tangeretin	citrus species (oranges, lemons, grapefruit, kumquat (golden orange of Corfu Islands)
Isoflavones	genistein, daidzein	soybean
Flavan-3-ols	catechin, epigallocatechin gallate	green tea, grapes, apples, beans, red wine, aronia fruits
Ellagitannins	punicagin, punicalagin	pomegranate
Anthocyanidins	cyanidin, malvidin, pelargonidin	blackberry, bilberry, strawberry, black currant, red currant, plums
**Non-flavonoids**		
Phenolcarboxylic	rosmarinic acid	oregano, thyme, peppermint, sage, basil
acids	caffeic acid, chlorogenic acid, cinnamic acid, p-coumaric acid	artichoke fruits, coffee, apples, plums
	ferulic acid	fennel (whole plant)
Stilbenes	resveratrol, piceatannol, pterostilbene,	red wine, red grapes
Lignans	lariciresinol, secoiolariciresinol, matairesinol	flaxseeds, whole grain cereals, cabbages, fresh green leaves, olives
Other compounds	hydroxytyrosol, oleocanthal, oleacein,	olive oil, olives
	curcumin	turmeric
	gingerols, shogaols	ginger
NON-PHENOLIC COMPOUNDS		
Carotenoids	α-carotene, β-carotene, lycopene, astaxanthin, lutein, zeaxanthin, fucoxanthin, crocetin	carrots, tomatoes, pumpkin, spinach, avocado, watermelon, saffron, kaki, seaweeds, endive, chicory leaves, dry beans, lentils, sea buckthorn fruits, citrus species
Terpenic compounds	ursolic acid, oleanolic acid	basil, sage, thyme
Phytosterols	stigmasterol, campesterol, β-sitosterol	white cabbage, zucchini, pumpkin seeds, oat, peanuts, sunflower seeds, seaweeds
Vitamin E	tocotrienols and tocopherols	nuts, almonds, pistachio, hazelnuts
Nitrogen compounds	piperine, capsaicin	black pepper, chili pepper
Organosulfur compounds	sulphoraphane, aliin, alicin, phenethyl isothiocyanate, triallyl disulfide	garlic, onion, broccoli
Omega-3/omega-6/omega-9 fatty acids	eicosapentaenoic acid (EPA), docosahexaenoic acid (DHA), oleic acid, α-linolenic acid, linoleic acid	fish (salmon, mackerel, anchovies, cod liver, tuna), shrimps, sardines, oysters, mussels, nuts, olive oil
Aromatic compounds	eugenol	clove buds
	cinnamaldehyde,	cinnamon
	thymoquinone,	black cumin seeds
Dietary fiber	inulin, fructo-oligosaccharides	leek, onion, sweet potatoes, whole grain cereals

**Table 4:** Outcomes regarding the effects of several dietary compounds in clinical studies.

Patient Condition	Dose of the Dietary Compound	Findings	Ref.
Curcumin			
Advanced metastatic cancer, including CRC, BC, PCa	100–300 mg/m^2^ liposomal curcumin (Lipocurc™)	All patients (*n* = 32) showed progressive disease and one patient showed stable disease In one PC patient, PSA was reduced from 649 to 355 ng/mL In one patient with CRC, CEA level was reduced from 18,542 to 6441 µg/L and CA19-9 from 18,105 to 13,238 U/mL More than 50% of the patients experienced side effects, such as anemia, hemolysis	[318]
Advanced, metastatic BC	6000 mg/day curcumin, for 7 days every 3 weeks, p.o. 100 mg/m^2^ docetaxel, every 3 weeks on day 1 for 6 cycles, i.v.	Out of 14 patients, 3 dose-limiting toxicities were observed Reduction in CEA marker	[319]
Lycopene			
Localized PCa	30 mg/day for 3 weeks, p.o.	Decreased tumor size, decreased PSA, and more negative resection margins in lycopene treated (*n* = 15) compared with control (*n* = 11) patients	[320]
Metastatic, castrate resistant PCa	21-day cycles of 75 mg/m^2^ docetaxel, plus lycopene 30 mg, p.o.	Treatment was overall well tolerated in the 13 patients treated; however, grade 3 or 4 neutropenia occurred in 4 patients and peripheral neuropathy in one patient The observed PSA response rate was 77% and the disease control rate was 92%, higher than values reported in the literature for docetaxel alone; however, this study had no control arm	[321]
Non-metastatic PC	21-day cycles of 75 mg/m^2^ docetaxel, androgen deprivation therapy, plus 30, 90, or 150 mg/day of synthetic lycopene, p.o.	Dose limiting toxicity at 150 mg/day in 1 out of 12 patients Lycopene improved the pharmacokinetics of docetaxel (increased AUC and C_max_)	[322]
Poly-unsaturated fatty acids (PUFA)			
CRC after elective surgery	0.8–1.5 g lipid emulsion/kg/day for 8 days (perioperative)	Well tolerated, a non-significant trend of lower proinflammatory markers in n-3 PUFA-enriched emulsion (*n* = 44) compared with control lipid emulsion (*n* = 41) was observed	[323]
Non-metastatic CRC	0.2 g lipid emulsion /kg/day for 2 days before surgery	Significantly more post-operative infectious complications in patients treated with n-3 PUFA (8/17 patients or 47%) compared with saline control (2/18 or 11%)	[324]
Solid cancers, predominantly BC	Taxol plus ~2 g n-3 PUFA per day, p.o.	Patients in n-3 PUFA administration group (*n* = 21) showed reduced incidence of paclitaxel-induced peripheral neuropathy, compared with patients in control group (*n* = 21)	[325]
BC stages I–IV	Paclitaxel (70–90 mg/m2) plus up to 4 g/day of n-3 PUFA ethyl esters, p.o.	No effect in reducing acute pain syndrome associated with paclitaxel in n-3 PUFA treated patients (*n* = 25) vs. placebo (*n* = 24)	[326]
Vitamin D			
CRC precursor lesions (sessile serrated adenomas or polyps)	1200 mg/day of elemental calcium, 1000 IU/day of vitamin D3 for 3 to 5 years	Calcium and vitamin D increased the risk of sessile serrated adenomas or polyps at 6–10 years after administration started	[327]
BC before surgery	400–10,000 IU/day vitamin D, between biopsy and surgery	Vitamin D decreased circulating 27-hydroxydroxycholesterol (27HC), a modulator of BC tumor growth positive for estrogen receptors (*n* = 29) The levels of vitamin D and the modulator of the estrogen receptors (27HD) were invers correlated	[328]
Advanced androgen-insensitive PCa	5–25 µg/mL paricalcitol (vitamin D) i.v., 3 times/week	Total of 18 patients Several large declines in PSA; however, not sustained 50% drop in serum PSA Paricalcitol was well tolerated with one significant hypercalcemia The levels of serum parathyroid hormone, negatively associated with survival, were reduced by paricalcitol	[329]
PCa after the surgery	4000 IU daily vitamin D3, 2 months prior to surgery	Gene expression involved in immune response and inflammation can be modulated by short administration of vitamin D3	[330]
Low-risk PCa	4000 IU daily vitamin D3 for 1 year	55% of the patients (24 out of 44) showed decrease in Gleason score, 11% no changes, and 34% increase in Gleason score	[331]
Vitamin A			
Metastatic CRC	0.5 mg/kg, 13-cis-retinoic acid, p.o., in combination with low-dose subcutaneous IL-2 as maintainance immunotherapy	Metastatic colorectal cancer patients (*n* = 40) were previously treated with induction chemotherapy Skin rash was observed in 29% and fever in 20% of the patients After 4 months, patients started to display increases in the level of lymphocytes (37%) and NK cells (81%), with the maximum difference between treatment and control group being observed after 2 years Significant improvements were observed in PFS and OS in the maintenance therapy versus the control group	[332]
Advanced or recurrent BC	45 mg/m^2^ all trans-retinoic acid, p.o. daily for 4 days, before paclitaxel treatment, repeated in 28 days cycles until progression or no longer tolerated	Pilot study comprising 17 patients. Grade 3 toxicity was mainly related to typical chemotherapy side effects, and no severe toxicities previously associated with paclitaxel were reported. Nausea and vomiting were reported in 2 patients and were presumably associated with protracted retinoic acid treatment. Overall, patients displayed a 76% clinical benefit. The response rate to the combination therapy was rather low, but the clinical efficacy was improved, when compared with paclitaxel alone studies. The time to progression and survival rates were similar to ones reported for paclitaxel alone	[333]
Metastatic PCa	Isotretinoin administered 1 mg/kg, p.o., 2x/day	37 patients randomized to add or not to add isotretinoin to antiandrogen treatment Isotretinoin was well tolerated with minor side effects such as cheilitis, skin dryness, and elevation of triglycerides No effect on PSA was observed during the 25 weeks of the therapy, or after one year, suggesting that isotretinoin has no negative effect on the reponse to hormone-ablative therapy	[334]

Legend: p.o., per os (orally); i.v., intravenous; PSA, prostate-specific antigen; CEA, carcinoembryonic antigen; PFS, progression free survival; OS, overall survival.

## References

[1] Visvader J.E., Lindeman G.J. (2012). Cancer stem cells: Current status and evolving complexities. Cell Stem Cell.

[2] Batlle E., Clevers H. (2017). Cancer stem cells revisited. Nat. Med..

[3] Prager B.C., Xie Q., Bao S., Rich J.N. (2019). Cancer Stem Cells: The Architects of the Tumor Ecosystem. Cell Stem Cell.

[4] Zakrzewski W., Dobrzynski M., Szymonowicz M., Rybak Z. (2019). Stem cells: Past, present, and future. Stem Cell Res. Ther..

[5] Plaks V., Kong N., Werb Z. (2015). The cancer stem cell niche: How essential is the niche in regulating stemness of tumor cells?. Cell Stem Cell.

[6] Nassar D., Blanpain C. (2016). Cancer Stem Cells: Basic Concepts and Therapeutic Implications. Annu. Rev. Pathol..

[7] Luo M., Li J.F., Yang Q., Zhang K., Wang Z.W., Zheng S., Zhou J.J. (2020). Stem cell quiescence and its clinical relevance. World J. Stem Cells.

[8] Paul R., Dorsey J.F., Fan Y. (2022). Cell plasticity, senescence, and quiescence in cancer stem cells: Biological and therapeutic implications. Pharmacol. Ther..

[9] Farhud D.D. (2015). Impact of Lifestyle on Health. Iran. J. Public Health.

[10] Viallon V., Freisling H., Matta K., Nannsen A.O., Dahm C.C., Tjonneland A., Eriksen A.K., Kaaks R., Katzke V.A., Schulze M.B. (2024). On the use of the healthy lifestyle index to investigate specific disease outcomes. Sci. Rep..

[11] Hu P., Zheng M., Huang J., Fan H.Y., Fan C.J., Ruan H.H., Yuan Y.S., Zhao W., Wang H.H.X., Deng H. (2022). Effect of healthy lifestyle index and lifestyle patterns on the risk of mortality: A community-based cohort study. Front. Med..

[12] Brivio F., Vigano A., Paterna A., Palena N., Greco A. (2023). Narrative Review and Analysis of the Use of “Lifestyle” in Health Psychology. Int. J. Environ. Res. Public Health.

[13] Sundermann M., Chielli D., Spell S. (2023). Nature As Medicine: The 7th (Unofficial) Pillar of Lifestyle Medicine. Am. J. Lifestyle Med..

[14] Chen Y., Wang X.Q., Zhang Q., Zhu J.Y., Li Y., Xie C.F., Li X.T., Wu J.S., Geng S.S., Zhong C.Y. (2017). (-)-Epigallocatechin-3-Gallate Inhibits Colorectal Cancer Stem Cells by Suppressing Wnt/beta-Catenin Pathway. Nutrients.

[15] Toden S., Tran H.M., Tovar-Camargo O.A., Okugawa Y., Goel A. (2016). Epigallocatechin-3-gallate targets cancer stem-like cells and enhances 5-fluorouracil chemosensitivity in colorectal cancer. Oncotarget.

[16] Lee J., Kim Y.S., Lee J., Heo S.C., Lee K.L., Choi S.W., Kim Y. (2016). Walnut Phenolic Extract and Its Bioactive Compounds Suppress Colon Cancer Cell Growth by Regulating Colon Cancer Stemness. Nutrients.

[17] Ahmed M., Ffrench-Constant C. (2016). Extracellular Matrix Regulation of Stem Cell Behavior. Curr. Stem Cell Rep..

[18] Manneken J.D., Currie P.D. (2023). Macrophage-stem cell crosstalk: Regulation of the stem cell niche. Development.

[19] Lakshmipathy U., Verfaillie C. (2005). Stem cell plasticity. Blood Rev..

[20] Zhu G., Hu J., Xi R. (2021). The cellular niche for intestinal stem cells: A team effort. Cell Regen..

[21] Kwon O.J., Xin L. (2014). Prostate epithelial stem and progenitor cells. Am. J. Clin. Exp. Urol..

[22] Takao T., Tsujimura A. (2008). Prostate stem cells: The niche and cell markers. Int. J. Urol..

[23] Karthaus W.R., Hofree M., Choi D., Linton E.L., Turkekul M., Bejnood A., Carver B., Gopalan A., Abida W., Laudone V. (2020). Regenerative potential of prostate luminal cells revealed by single-cell analysis. Science.

[24] Celia-Terrassa T. (2018). Mammary Stem Cells and Breast Cancer Stem Cells: Molecular Connections and Clinical Implications. Biomedicines.

[25] Rios A.C., Fu N.Y., Lindeman G.J., Visvader J.E. (2014). In situ identification of bipotent stem cells in the mammary gland. Nature.

[26] Huang E.H., Hynes M.J., Zhang T., Ginestier C., Dontu G., Appelman H., Fields J.Z., Wicha M.S., Boman B.M. (2009). Aldehyde dehydrogenase 1 is a marker for normal and malignant human colonic stem cells (SC) and tracks SC overpopulation during colon tumorigenesis. Cancer Res..

[27] Ginestier C., Hur M.H., Charafe-Jauffret E., Monville F., Dutcher J., Brown M., Jacquemier J., Viens P., Kleer C.G., Liu S. (2007). ALDH1 is a marker of normal and malignant human mammary stem cells and a predictor of poor clinical outcome. Cell Stem Cell.

[28] Montgomery R.K., Shivdasani R.A. (2009). Prominin1 (CD133) as an intestinal stem cell marker: Promise and nuance. Gastroenterology.

[29] Anderson L.H., Boulanger C.A., Smith G.H., Carmeliet P., Watson C.J. (2011). Stem cell marker prominin-1 regulates branching morphogenesis, but not regenerative capacity, in the mammary gland. Dev. Dyn..

[30] Shackleton M., Vaillant F., Simpson K.J., Stingl J., Smyth G.K., Asselin-Labat M.L., Wu L., Lindeman G.J., Visvader J.E. (2006). Generation of a functional mammary gland from a single stem cell. Nature.

[31] Barclay W.W., Axanova L.S., Chen W., Romero L., Maund S.L., Soker S., Lees C.J., Cramer S.D. (2008). Characterization of adult prostatic progenitor/stem cells exhibiting self-renewal and multilineage differentiation. Stem Cells.

[32] Stingl J., Eirew P., Ricketson I., Shackleton M., Vaillant F., Choi D., Li H.I., Eaves C.J. (2006). Purification and unique properties of mammary epithelial stem cells. Nature.

[33] Munoz J., Stange D.E., Schepers A.G., van de Wetering M., Koo B.K., Itzkovitz S., Volckmann R., Kung K.S., Koster J., Radulescu S. (2012). The Lgr5 intestinal stem cell signature: Robust expression of proposed quiescent ‘+4’ cell markers. EMBO J..

[34] van der Flier L.G., Haegebarth A., Stange D.E., van de Wetering M., Clevers H. (2009). OLFM4 is a robust marker for stem cells in human intestine and marks a subset of colorectal cancer cells. Gastroenterology.

[35] Welm B.E., Tepera S.B., Venezia T., Graubert T.A., Rosen J.M., Goodell M.A. (2002). Sca-1(pos) cells in the mouse mammary gland represent an enriched progenitor cell population. Dev. Biol..

[36] Sangiorgi E., Capecchi M.R. (2008). Bmi1 is expressed in vivo in intestinal stem cells. Nat. Genet..

[37] Meyfour A., Pahlavan S., Mirzaei M., Krijgsveld J., Baharvand H., Salekdeh G.H. (2021). The quest of cell surface markers for stem cell therapy. Cell Mol. Life Sci..

[38] Watson C.J. (2021). How should we define mammary stem cells?. Trends Cell Biol..

[39] Espinosa-Sanchez A., Suarez-Martinez E., Sanchez-Diaz L., Carnero A. (2020). Therapeutic Targeting of Signaling Pathways Related to Cancer Stemness. Front. Oncol..

[40] Tan S.H., Barker N. (2018). Wnt Signaling in Adult Epithelial Stem Cells and Cancer. Prog. Mol. Biol. Transl. Sci..

[41] Kahn M. (2018). Wnt Signaling in Stem Cells and Cancer Stem Cells: A Tale of Two Coactivators. Prog. Mol. Biol. Transl. Sci..

[42] Katagiri T., Watabe T. (2016). Bone Morphogenetic Proteins. Cold Spring Harb. Perspect. Biol..

[43] Qi Z., Li Y., Zhao B., Xu C., Liu Y., Li H., Zhang B., Wang X., Yang X., Xie W. (2017). BMP restricts stemness of intestinal Lgr5(+) stem cells by directly suppressing their signature genes. Nat. Commun..

[44] Bigas A., Porcheri C. (2018). Notch and Stem Cells. Adv. Exp. Med. Biol..

[45] Clevers H., Loh K.M., Nusse R. (2014). Stem cell signaling. An integral program for tissue renewal and regeneration: Wnt signaling and stem cell control. Science.

[46] Albrecht L.V., Tejeda-Munoz N., De Robertis E.M. (2021). Cell Biology of Canonical Wnt Signaling. Annu. Rev. Cell Dev. Biol..

[47] Zhou B., Lin W., Long Y., Yang Y., Zhang H., Wu K., Chu Q. (2022). Notch signaling pathway: Architecture, disease, and therapeutics. Signal Transduct. Target. Ther..

[48] Pellegrinet L., Rodilla V., Liu Z., Chen S., Koch U., Espinosa L., Kaestner K.H., Kopan R., Lewis J., Radtke F. (2011). Dll1- and dll4-mediated notch signaling are required for homeostasis of intestinal stem cells. Gastroenterology.

[49] Riccio O., van Gijn M.E., Bezdek A.C., Pellegrinet L., van Es J.H., Zimber-Strobl U., Strobl L.J., Honjo T., Clevers H., Radtke F. (2008). Loss of intestinal crypt progenitor cells owing to inactivation of both Notch1 and Notch2 is accompanied by derepression of CDK inhibitors p27Kip1 and p57Kip2. EMBO Rep..

[50] Xing W., Yang J., Zheng Y., Yao L., Peng X., Chen Y., Yang C. (2024). The Role of the Notch Signaling Pathway in the Differentiation of Human Umbilical Cord-Derived Mesenchymal Stem Cells. Front. Biosci..

[51] Duncan A.W., Rattis F.M., DiMascio L.N., Congdon K.L., Pazianos G., Zhao C., Yoon K., Cook J.M., Willert K., Gaiano N. (2005). Integration of Notch and Wnt signaling in hematopoietic stem cell maintenance. Nat. Immunol..

[52] Jiang L., Tian J., Yang J., Luo R., Zhang Y., Shao C., Guo B., Wu X., Dan J., Luo Y. (2024). p21 Regulates Wnt-Notch balance via DREAM/MMB/Rb-E2F1 and maintains intestinal stem cell homeostasis. Cell Death Discov..

[53] Centonze A., Lin S., Tika E., Sifrim A., Fioramonti M., Malfait M., Song Y., Wuidart A., Van Herck J., Dannau A. (2020). Heterotypic cell-cell communication regulates glandular stem cell multipotency. Nature.

[54] Sato T., van Es J.H., Snippert H.J., Stange D.E., Vries R.G., van den Born M., Barker N., Shroyer N.F., van de Wetering M., Clevers H. (2011). Paneth cells constitute the niche for Lgr5 stem cells in intestinal crypts. Nature.

[55] Zhang Y., Que J. (2020). BMP Signaling in Development, Stem Cells, and Diseases of the Gastrointestinal Tract. Annu. Rev. Physiol..

[56] Wu M., Wu S., Chen W., Li Y.P. (2024). The roles and regulatory mechanisms of TGF-beta and BMP signaling in bone and cartilage development, homeostasis and disease. Cell Res..

[57] Alison M.R., Poulsom R., Forbes S., Wright N.A. (2002). An introduction to stem cells. J. Pathol..

[58] Gupta P.B., Fillmore C.M., Jiang G., Shapira S.D., Tao K., Kuperwasser C., Lander E.S. (2011). Stochastic state transitions give rise to phenotypic equilibrium in populations of cancer cells. Cell.

[59] Nair N., Calle A.S., Zahra M.H., Prieto-Vila M., Oo A.K.K., Hurley L., Vaidyanath A., Seno A., Masuda J., Iwasaki Y. (2017). A cancer stem cell model as the point of origin of cancer-associated fibroblasts in tumor microenvironment. Sci. Rep..

[60] Su S., Chen J., Yao H., Liu J., Yu S., Lao L., Wang M., Luo M., Xing Y., Chen F. (2018). CD10(+)GPR77(+) Cancer-Associated Fibroblasts Promote Cancer Formation and Chemoresistance by Sustaining Cancer Stemness. Cell.

[61] Radharani N.N.V., Yadav A.S., Nimma R., Kumar T.V.S., Bulbule A., Chanukuppa V., Kumar D., Patnaik S., Rapole S., Kundu G.C. (2022). Tumor-associated macrophage derived IL-6 enriches cancer stem cell population and promotes breast tumor progression via Stat-3 pathway. Cancer Cell Int..

[62] Lu H., Clauser K.R., Tam W.L., Frose J., Ye X., Eaton E.N., Reinhardt F., Donnenberg V.S., Bhargava R., Carr S.A. (2014). A breast cancer stem cell niche supported by juxtacrine signalling from monocytes and macrophages. Nat. Cell Biol..

[63] Osman A., Oze M., Afify S.M., Hassan G., El-Ghlban S., Nawara H.M., Fu X., Zahra M.H., Seno A., Winer I. (2020). Tumor-associated macrophages derived from cancer stem cells. Acta Histochem..

[64] Phi L.T.H., Sari I.N., Yang Y.G., Lee S.H., Jun N., Kim K.S., Lee Y.K., Kwon H.Y. (2018). Cancer Stem Cells (CSCs) in Drug Resistance and their Therapeutic Implications in Cancer Treatment. Stem Cells Int..

[65] Marzagalli M., Fontana F., Raimondi M., Limonta P. (2021). Cancer Stem Cells-Key Players in Tumor Relapse. Cancers.

[66] Meyer M.J., Fleming J.M., Lin A.F., Hussnain S.A., Ginsburg E., Vonderhaar B.K. (2010). CD44posCD49fhiCD133/2hi defines xenograft-initiating cells in estrogen receptor-negative breast cancer. Cancer Res..

[67] de Beca F.F., Caetano P., Gerhard R., Alvarenga C.A., Gomes M., Paredes J., Schmitt F. (2013). Cancer stem cells markers CD44, CD24 and ALDH1 in breast cancer special histological types. J. Clin. Pathol..

[68] Leis O., Eguiara A., Lopez-Arribillaga E., Alberdi M.J., Hernandez-Garcia S., Elorriaga K., Pandiella A., Rezola R., Martin A.G. (2012). Sox2 expression in breast tumours and activation in breast cancer stem cells. Oncogene.

[69] Collins A.T., Maitland N.J. (2006). Prostate cancer stem cells. Eur. J. Cancer.

[70] Collins A.T., Berry P.A., Hyde C., Stower M.J., Maitland N.J. (2005). Prospective identification of tumorigenic prostate cancer stem cells. Cancer Res..

[71] Lawson D.A., Zong Y., Memarzadeh S., Xin L., Huang J., Witte O.N. (2010). Basal epithelial stem cells are efficient targets for prostate cancer initiation. Proc. Natl. Acad. Sci. USA.

[72] Matsika A., Srinivasan B., Day C., Mader S.A., Kiernan D.M., Broomfield A., Fu J., Hooper J.D., Kench J.G., Samaratunga H. (2015). Cancer stem cell markers in prostate cancer: An immunohistochemical study of ALDH1, SOX2 and EZH2. Pathology.

[73] Ricci-Vitiani L., Lombardi D.G., Pilozzi E., Biffoni M., Todaro M., Peschle C., De Maria R. (2007). Identification and expansion of human colon-cancer-initiating cells. Nature.

[74] Gupta B., Das P., Ghosh S., Manhas J., Sen S., Pal S., Sahni P., Upadhyay A.D., Panda S.K., Gupta S.D. (2017). Identification of High-Risk Aberrant Crypt Foci and Mucin-Depleted Foci in the Human Colon With Study of Colon Cancer Stem Cell Markers. Clin. Color. Cancer.

[75] Haraguchi N., Ishii H., Mimori K., Ohta K., Uemura M., Nishimura J., Hata T., Takemasa I., Mizushima T., Yamamoto H. (2013). CD49f-positive cell population efficiently enriches colon cancer-initiating cells. Int. J. Oncol..

[76] Glumac P.M., LeBeau A.M. (2018). The role of CD133 in cancer: A concise review. Clin. Transl. Med..

[77] Skandalis S.S., Karalis T.T., Chatzopoulos A., Karamanos N.K. (2019). Hyaluronan-CD44 axis orchestrates cancer stem cell functions. Cell Signal..

[78] Wei Y., Li Y., Chen Y., Liu P., Huang S., Zhang Y., Sun Y., Wu Z., Hu M., Wu Q. (2022). ALDH1: A potential therapeutic target for cancer stem cells in solid tumors. Front. Oncol..

[79] Manni W., Min W. (2022). Signaling pathways in the regulation of cancer stem cells and associated targeted therapy. MedComm (2020).

[80] Fearon E.R. (2011). Molecular genetics of colorectal cancer. Annu. Rev. Pathol..

[81] Seshagiri S., Stawiski E.W., Durinck S., Modrusan Z., Storm E.E., Conboy C.B., Chaudhuri S., Guan Y., Janakiraman V., Jaiswal B.S. (2012). Recurrent R-spondin fusions in colon cancer. Nature.

[82] Giannakis M., Hodis E., Jasmine Mu X., Yamauchi M., Rosenbluh J., Cibulskis K., Saksena G., Lawrence M.S., Qian Z.R., Nishihara R. (2014). RNF43 is frequently mutated in colorectal and endometrial cancers. Nat. Genet..

[83] Carballo G.B., Honorato J.R., de Lopes G.P.F., Spohr T. (2018). A highlight on Sonic hedgehog pathway. Cell Commun. Signal..

[84] Karhadkar S.S., Bova G.S., Abdallah N., Dhara S., Gardner D., Maitra A., Isaacs J.T., Berman D.M., Beachy P.A. (2004). Hedgehog signalling in prostate regeneration, neoplasia and metastasis. Nature.

[85] Liu S., Dontu G., Mantle I.D., Patel S., Ahn N.S., Jackson K.W., Suri P., Wicha M.S. (2006). Hedgehog signaling and Bmi-1 regulate self-renewal of normal and malignant human mammary stem cells. Cancer Res..

[86] Bray S.J. (2006). Notch signalling: A simple pathway becomes complex. Nat. Rev. Mol. Cell Biol..

[87] Ranganathan P., Weaver K.L., Capobianco A.J. (2011). Notch signalling in solid tumours: A little bit of everything but not all the time. Nat. Rev. Cancer.

[88] Stylianou S., Clarke R.B., Brennan K. (2006). Aberrant activation of notch signaling in human breast cancer. Cancer Res..

[89] Sethi N., Dai X., Winter C.G., Kang Y. (2011). Tumor-derived JAGGED1 promotes osteolytic bone metastasis of breast cancer by engaging notch signaling in bone cells. Cancer Cell.

[90] Sikandar S.S., Pate K.T., Anderson S., Dizon D., Edwards R.A., Waterman M.L., Lipkin S.M. (2010). NOTCH signaling is required for formation and self-renewal of tumor-initiating cells and for repression of secretory cell differentiation in colon cancer. Cancer Res..

[91] Tyagi A., Sharma A.K., Damodaran C. (2020). A Review on Notch Signaling and Colorectal Cancer. Cells.

[92] Lu J., Ye X., Fan F., Xia L., Bhattacharya R., Bellister S., Tozzi F., Sceusi E., Zhou Y., Tachibana I. (2013). Endothelial cells promote the colorectal cancer stem cell phenotype through a soluble form of Jagged-1. Cancer Cell.

[93] Zhou W., Yan K., Xi Q. (2023). BMP signaling in cancer stemness and differentiation. Cell Regen..

[94] Diehn M., Cho R.W., Lobo N.A., Kalisky T., Dorie M.J., Kulp A.N., Qian D., Lam J.S., Ailles L.E., Wong M. (2009). Association of reactive oxygen species levels and radioresistance in cancer stem cells. Nature.

[95] Vassalli G. (2019). Aldehyde Dehydrogenases: Not Just Markers, but Functional Regulators of Stem Cells. Stem Cells Int..

[96] Tuy K., Rickenbacker L., Hjelmeland A.B. (2021). Reactive oxygen species produced by altered tumor metabolism impacts cancer stem cell maintenance. Redox Biol..

[97] Liskova A., Kubatka P., Samec M., Zubor P., Mlyncek M., Bielik T., Samuel S.M., Zulli A., Kwon T.K., Busselberg D. (2019). Dietary Phytochemicals Targeting Cancer Stem Cells. Molecules.

[98] Elmaliklis I.-N., Miserli E., Filipatou M., Tsikouras I., Dimou C., Koutelidakis A. (2020). Association of Mediterranean Diet Adherence, Functional Food Consumption and Anthropometric Characteristics with Anxiety and Depression Indexes in a Sample of Healthy Greek Adults: A Cross-Sectional Study. Psychiatry Int..

[99] Mentella M.C., Scaldaferri F., Ricci C., Gasbarrini A., Miggiano G.A.D. (2019). Cancer and Mediterranean Diet: A Review. Nutrients.

[100] Ganesan K., Jayachandran M., Xu B. (2020). Diet-Derived Phytochemicals Targeting Colon Cancer Stem Cells and Microbiota in Colorectal Cancer. Int. J. Mol. Sci..

[101] Prajapati K.S., Gupta S., Kumar S. (2022). Targeting Breast Cancer-Derived Stem Cells by Dietary Phytochemicals: A Strategy for Cancer Prevention and Treatment. Cancers.

[102] Sanchez-Sanchez M.L., Garcia-Vigara A., Hidalgo-Mora J.J., Garcia-Perez M.A., Tarin J., Cano A. (2020). Mediterranean diet and health: A systematic review of epidemiological studies and intervention trials. Maturitas.

[103] Aguilera Y., Martín-Cabrejas M.A., González de Mejía E. (2015). Phenolic compounds in fruits and beverages consumed as part of the mediterranean diet: Their role in prevention of chronic diseases. Phytochem. Rev..

[104] Godos J., Marventano S., Mistretta A., Galvano F., Grosso G. (2017). Dietary sources of polyphenols in the Mediterranean healthy Eating, Aging and Lifestyle (MEAL) study cohort. Int. J. Food Sci. Nutr..

[105] Petreska J., Stefova M., Ferreres F., Moreno D.A., Tomás-Barberán F.A., Stefkov G., Kulevanova S., Gil-Izquierdo A. (2011). Dietary Burden of Phenolics per Serving of “Mountain Tea” (Sideritis) from Macedonia and Correlation to Antioxidant Activity. Nat. Prod. Commun..

[106] Mazurakova A., Koklesova L., Vybohova D., Samec M., Kudela E., Biringer K., Sudomova M., Hassan S.T.S., Kello M., Busselberg D. (2023). Therapy-resistant breast cancer in focus: Clinically relevant mitigation by flavonoids targeting cancer stem cells. Front. Pharmacol..

[107] Su Q., Rowley K.G., Itsiopoulos C., O’Dea K. (2002). Identification and quantitation of major carotenoids in selected components of the Mediterranean diet: Green leafy vegetables, figs and olive oil. Eur. J. Clin. Nutr..

[108] EL-Qudah J.M. (2014). Estimation of Carotenoid Contents of Selected Mediterranean Legumes by HPLC. World J. Med. Sci..

[109] Hoffman R., Gerber M. (2015). Food Processing and the Mediterranean Diet. Nutrients.

[110] Naureen Z., Bonetti G., Medori M.C., Aquilanti B., Velluti V., Matera G., Iaconelli A., Bertelli M. (2022). Foods of the Mediterranean diet: Garlic and Mediterranean legumes. J. Prev. Med. Hyg..

[111] Talib W.H., AlHur M.J., Al Naimat S., Ahmad R.E., Al-Yasari A.H., Al-Dalaeen A., Thiab S., Mahmod A.I. (2022). Anticancer Effect of Spices Used in Mediterranean Diet: Preventive and Therapeutic Potentials. Front. Nutr..

[112] Merra G., Noce A., Marrone G., Cintoni M., Tarsitano M.G., Capacci A., De Lorenzo A. (2020). Influence of Mediterranean Diet on Human Gut Microbiota. Nutrients.

[113] Hernandez-Lopez I., Ortiz-Sola J., Alamprese C., Barros L., Shelef O., Basheer L., Rivera A., Abadias M., Aguilo-Aguayo I. (2022). Valorization of Local Legumes and Nuts as Key Components of the Mediterranean Diet. Foods.

[114] Sohn S.I., Rathinapriya P., Balaji S., Jaya Balan D., Swetha T.K., Durgadevi R., Alagulakshmi S., Singaraj P., Pandian S. (2021). Phytosterols in Seaweeds: An Overview on Biosynthesis to Biomedical Applications. Int. J. Mol. Sci..

[115] Wang M., Firrman J., Liu L., Yam K. (2019). A Review on Flavonoid Apigenin: Dietary Intake, ADME, Antimicrobial Effects, and Interactions with Human Gut Microbiota. Biomed. Res. Int..

[116] Rauf A., Wilairatana P., Joshi P.B., Ahmad Z., Olatunde A., Hafeez N., Hemeg H.A., Mubarak M.S. (2024). Revisiting luteolin: An updated review on its anticancer potential. Heliyon.

[117] Li Y.W., Xu J., Zhu G.Y., Huang Z.J., Lu Y., Li X.Q., Wang N., Zhang F.X. (2018). Apigenin suppresses the stem cell-like properties of triple-negative breast cancer cells by inhibiting YAP/TAZ activity. Cell Death Discov..

[118] Sharma A., Sinha S., Keswani H., Shrivastava N. (2022). Kaempferol and Apigenin suppresses the stemness properties of TNBC cells by modulating Sirtuins. Mol. Divers..

[119] Vuoso D.C., D’Angelo S., Ferraro R., Caserta S., Guido S., Cammarota M., Porcelli M., Cacciapuoti G. (2020). Annurca apple polyphenol extract promotes mesenchymal-to-epithelial transition and inhibits migration in triple-negative breast cancer cells through ROS/JNK signaling. Sci. Rep..

[120] Ma H., Yue G.G., Lee J.K., Gao S., Yuen K.K., Cheng W., Li X., Lau C.B. (2024). Scutellarin, a flavonoid compound from Scutellaria barbata, suppresses growth of breast cancer stem cells in vitro and in tumor-bearing mice. Phytomedicine.

[121] Mei X., Ouyang H., Zhang H., Jia W., Lu B., Zhang J., Ji L. (2023). Scutellarin suppresses the metastasis of triple-negative breast cancer via targeting TNFalpha/TNFR2-RUNX1-triggered G-CSF expression in endothelial cells. Biochem. Pharmacol..

[122] Erdogan S., Turkekul K., Dibirdik I., Doganlar Z.B., Doganlar O., Bilir A. (2020). Midkine silencing enhances the anti-prostate cancer stem cell activity of the flavone apigenin: Cooperation on signaling pathways regulated by ERK, p38, PTEN, PARP, and NF-kappaB. Investig. New Drugs.

[123] Cook M.T., Liang Y., Besch-Williford C., Goyette S., Mafuvadze B., Hyder S.M. (2015). Luteolin inhibits progestin-dependent angiogenesis, stem cell-like characteristics, and growth of human breast cancer xenografts. Springerplus.

[124] Tsai K.J., Tsai H.Y., Tsai C.C., Chen T.Y., Hsieh T.H., Chen C.L., Mbuyisa L., Huang Y.B., Lin M.W. (2021). Luteolin Inhibits Breast Cancer Stemness and Enhances Chemosensitivity through the Nrf2-Mediated Pathway. Molecules.

[125] Tsai P.H., Cheng C.H., Lin C.Y., Huang Y.T., Lee L.T., Kandaswami C.C., Lin Y.C., Lee K.P., Hung C.C., Hwang J.J. (2016). Dietary Flavonoids Luteolin and Quercetin Suppressed Cancer Stem Cell Properties and Metastatic Potential of Isolated Prostate Cancer Cells. Anticancer Res..

[126] Li S., Zhao Q., Wang B., Yuan S., Wang X., Li K. (2018). Quercetin reversed MDR in breast cancer cells through down-regulating P-gp expression and eliminating cancer stem cells mediated by YB-1 nuclear translocation. Phytother. Res..

[127] Li X., Zhou N., Wang J., Liu Z., Wang X., Zhang Q., Liu Q., Gao L., Wang R. (2018). Quercetin suppresses breast cancer stem cells (CD44(+)/CD24(-)) by inhibiting the PI3K/Akt/mTOR-signaling pathway. Life Sci..

[128] Azizi E., Fouladdel S., Komeili Movahhed T., Modaresi F., Barzegar E., Ghahremani M.H., Ostad S.N., Atashpour S. (2022). Quercetin Effects on Cell Cycle Arrest and Apoptosis and Doxorubicin Activity in T47D Cancer Stem Cells. Asian Pac. J. Cancer Prev..

[129] Turkekul K., Erdogan S. (2023). Potent Suppression of Prostate Cancer Cell Growth and Eradication of Cancer Stem Cells by CD44-targeted Nanoliposome-quercetin Nanoparticles. J. Cancer Prev..

[130] Baruah M.M., Khandwekar A.P., Sharma N. (2016). Quercetin modulates Wnt signaling components in prostate cancer cell line by inhibiting cell viability, migration, and metastases. Tumour Biol..

[131] Erdogan S., Turkekul K., Dibirdik I., Doganlar O., Doganlar Z.B., Bilir A., Oktem G. (2018). Midkine downregulation increases the efficacy of quercetin on prostate cancer stem cell survival and migration through PI3K/AKT and MAPK/ERK pathway. Biomed. Pharmacother..

[132] Nandi S.K., Pradhan A., Das B., Das B., Basu S., Mallick B., Dutta A., Sarkar D.K., Mukhopadhyay A., Mukhopadhyay S. (2022). Kaempferol attenuates viability of ex-vivo cultured post-NACT breast tumor explants through downregulation of p53 induced stemness, inflammation and apoptosis evasion pathways. Pathol. Res. Pract..

[133] Nandi S.K., Roychowdhury T., Chattopadhyay S., Basu S., Chatterjee K., Choudhury P., Banerjee N., Saha P., Mukhopadhyay S., Mukhopadhyay A. (2022). Deregulation of the CD44-NANOG-MDR1 associated chemoresistance pathways of breast cancer stem cells potentiates the anti-cancer effect of Kaempferol in synergism with Verapamil. Toxicol. Appl. Pharmacol..

[134] Song X., Tan L., Wang M., Ren C., Guo C., Yang B., Ren Y., Cao Z., Li Y., Pei J. (2021). Myricetin: A review of the most recent research. Biomed. Pharmacother..

[135] Hu J., Zhang Y., Jiang X., Zhang H., Gao Z., Li Y., Fu R., Li L., Li J., Cui H. (2019). ROS-mediated activation and mitochondrial translocation of CaMKII contributes to Drp1-dependent mitochondrial fission and apoptosis in triple-negative breast cancer cells by isorhamnetin and chloroquine. J. Exp. Clin. Cancer Res..

[136] Amalina N.D., Salsabila I.A., Zulfin U.M., Jenie R.I., Meiyanto E. (2023). In vitro synergistic effect of hesperidin and doxorubicin downregulates epithelial-mesenchymal transition in highly metastatic breast cancer cells. J. Egypt. Natl. Canc Inst..

[137] Hermawan A., Khumaira A., Ikawati M., Putri H., Jenie R.I., Angraini S.M., Muflikhasari H.A. (2021). Identification of key genes of hesperidin in inhibition of breast cancer stem cells by functional network analysis. Comput. Biol. Chem..

[138] Chen S.J., Lu J.H., Lin C.C., Zeng S.W., Chang J.F., Chung Y.C., Chang H., Hsu C.P. (2023). Synergistic Chemopreventive Effects of a Novel Combined Plant Extract Comprising Gallic Acid and Hesperidin on Colorectal Cancer. Curr. Issues Mol. Biol..

[139] Zamanian M.Y., Golmohammadi M., Abdullaev B., Garcia M.O., Alazbjee A.A.A., Kumar A., Mohaamed S.S., Hussien B.M., Khalaj F., Hodaei S.M. (2024). A narrative review on therapeutic potential of naringenin in colorectal cancer: Focusing on molecular and biochemical processes. Cell Biochem. Funct..

[140] Turdo A., Glaviano A., Pepe G., Calapa F., Raimondo S., Fiori M.E., Carbone D., Basilicata M.G., Di Sarno V., Ostacolo C. (2021). Nobiletin and Xanthohumol Sensitize Colorectal Cancer Stem Cells to Standard Chemotherapy. Cancers.

[141] Ko Y.C., Choi H.S., Liu R., Kim J.H., Kim S.L., Yun B.S., Lee D.S. (2020). Inhibitory Effects of Tangeretin, A Citrus Peel-Derived Flavonoid, on Breast Cancer Stem Cell Formation through Suppression of Stat3 Signaling. Molecules.

[142] Zhu W.B., Xiao N., Liu X.J. (2018). Dietary flavonoid tangeretin induces reprogramming of epithelial to mesenchymal transition in prostate cancer cells by targeting the PI3K/Akt/mTOR signaling pathway. Oncol. Lett..

[143] Fan P., Fan S., Wang H., Mao J., Shi Y., Ibrahim M.M., Ma W., Yu X., Hou Z., Wang B. (2013). Genistein decreases the breast cancer stem-like cell population through Hedgehog pathway. Stem Cell Res. Ther..

[144] Sekar V., Anandasadagopan S.K., Ganapasam S. (2016). Genistein regulates tumor microenvironment and exhibits anticancer effect in dimethyl hydrazine-induced experimental colon carcinogenesis. Biofactors.

[145] Kwon H., Kim Y., Kim J.H. (2024). A combination of myokines and genistein suppresses cancer stemness in MCF-7 human breast cancer cells. Nutr. Res. Pract..

[146] Montales M.T., Rahal O.M., Kang J., Rogers T.J., Prior R.L., Wu X., Simmen R.C. (2012). Repression of mammosphere formation of human breast cancer cells by soy isoflavone genistein and blueberry polyphenolic acids suggests diet-mediated targeting of cancer stem-like/progenitor cells. Carcinogenesis.

[147] Zhang L., Li L., Jiao M., Wu D., Wu K., Li X., Zhu G., Yang L., Wang X., Hsieh J.T. (2012). Genistein inhibits the stemness properties of prostate cancer cells through targeting Hedgehog-Gli1 pathway. Cancer Lett..

[148] Zhang Q., Cheng G., Qiu H., Wang Y., Wang J., Xu H., Zhang T., Liu L., Tao Y., Ren Z. (2017). Expression of prostate stem cell antigen is downregulated during flavonoid-induced cytotoxicity in prostate cancer cells. Exp. Ther. Med..

[149] Shin P.K., Zoh Y., Choi J., Kim M.S., Kim Y., Choi S.W. (2019). Walnut phenolic extracts reduce telomere length and telomerase activity in a colon cancer stem cell model. Nutr. Res. Pract..

[150] Choi H.S., Kim J.H., Kim S.L., Deng H.Y., Lee D., Kim C.S., Yun B.S., Lee D.S. (2018). Catechol derived from aronia juice through lactic acid bacteria fermentation inhibits breast cancer stem cell formation via modulation Stat3/IL-6 signaling pathway. Mol. Carcinog..

[151] Shimizu M., Fukutomi Y., Ninomiya M., Nagura K., Kato T., Araki H., Suganuma M., Fujiki H., Moriwaki H. (2008). Green tea extracts for the prevention of metachronous colorectal adenomas: A pilot study. Cancer Epidemiol. Biomark. Prev..

[152] Tang S.N., Singh C., Nall D., Meeker D., Shankar S., Srivastava R.K. (2010). The dietary bioflavonoid quercetin synergizes with epigallocathechin gallate (EGCG) to inhibit prostate cancer stem cell characteristics, invasion, migration and epithelial-mesenchymal transition. J. Mol. Signal..

[153] Nunez-Sanchez M.A., Karmokar A., Gonzalez-Sarrias A., Garcia-Villalba R., Tomas-Barberan F.A., Garcia-Conesa M.T., Brown K., Espin J.C. (2016). In vivo relevant mixed urolithins and ellagic acid inhibit phenotypic and molecular colon cancer stem cell features: A new potentiality for ellagitannin metabolites against cancer. Food Chem. Toxicol..

[154] Ahmed H.H., El-Abhar H.S., Hassanin E.A.K., Abdelkader N.F., Shalaby M.B. (2017). Punica granatum suppresses colon cancer through downregulation of Wnt/β-Catenin in rat model. Rev. Bras. Farmacogn..

[155] Dai Z., Nair V., Khan M., Ciolino H.P. (2010). Pomegranate extract inhibits the proliferation and viability of MMTV-Wnt-1 mouse mammary cancer stem cells in vitro. Oncol. Rep..

[156] Bagheri M., Fazli M., Saeednia S., Kor A., Ahmadiankia N. (2018). Pomegranate peel extract inhibits expression of Î²-catenin, epithelial mesenchymal transition, and metastasis in triple negative breast cancer cells. Cell. Mol. Biol..

[157] Nallanthighal S., Elmaliki K.M., Reliene R. (2017). Pomegranate Extract Alters Breast Cancer Stem Cell Properties in Association with Inhibition of Epithelial-to-Mesenchymal Transition. Nutr. Cancer.

[158] Chaves F.M., Pavan I.C.B., da Silva L.G.S., de Freitas L.B., Rostagno M.A., Antunes A.E.C., Bezerra R.M.N., Simabuco F.M. (2020). Pomegranate Juice and Peel Extracts are Able to Inhibit Proliferation, Migration and Colony Formation of Prostate Cancer Cell Lines and Modulate the Akt/mTOR/S6K Signaling Pathway. Plant Foods Hum. Nutr..

[159] Charepalli V., Reddivari L., Radhakrishnan S., Vadde R., Agarwal R., Vanamala J.K. (2015). Anthocyanin-containing purple-fleshed potatoes suppress colon tumorigenesis via elimination of colon cancer stem cells. J. Nutr. Biochem..

[160] Mortoglou M., Lian M., Miralles F., Dart D.A., Uysal-Onganer P. (2024). miR-210 Mediated Hypoxic Responses in Pancreatic Ductal Adenocarcinoma. ACS Omega.

[161] Huang S., Guo W., Tang Y., Ren D., Zou X., Peng X. (2012). miR-143 and miR-145 inhibit stem cell characteristics of PC-3 prostate cancer cells. Oncol. Rep..

[162] Vuong T., Mallet J.F., Ouzounova M., Rahbar S., Hernandez-Vargas H., Herceg Z., Matar C. (2016). Role of a polyphenol-enriched preparation on chemoprevention of mammary carcinoma through cancer stem cells and inflammatory pathways modulation. J. Transl. Med..

[163] Borras-Linares I., Perez-Sanchez A., Lozano-Sanchez J., Barrajon-Catalan E., Arraez-Roman D., Cifuentes A., Micol V., Carretero A.S. (2015). A bioguided identification of the active compounds that contribute to the antiproliferative/cytotoxic effects of rosemary extract on colon cancer cells. Food Chem. Toxicol..

[164] Wang W., Zhang Y., Huang X., Li D., Lin Q., Zhuang H., Li H. (2024). The role of the miR-30a-5p/BCL2L11 pathway in rosmarinic acid-induced apoptosis in MDA-MB-231-derived breast cancer stem-like cells. Front. Pharmacol..

[165] Raad C., Raad A., Pandey S. (2024). Green Tea Leaves and Rosemary Extracts Selectively Induce Cell Death in Triple-Negative Breast Cancer Cells and Cancer Stem Cells and Enhance the Efficacy of Common Chemotherapeutics. Evid. Based Complement. Altern. Med..

[166] Kubatka P., Kello M., Kajo K., Kruzliak P., Vybohova D., Mojzis J., Adamkov M., Fialova S., Veizerova L., Zulli A. (2017). Oregano demonstrates distinct tumour-suppressive effects in the breast carcinoma model. Eur. J. Nutr..

[167] Trikka F.A., Michailidou S., Makris A.M., Argiriou A. (2019). Biochemical Fingerprint of Greek Sideritis spp.: Implications for Potential Drug Discovery and Advanced Breeding Strategies. Med. Aromat. Plants.

[168] Soltanian S., Riahirad H., Pabarja A., Jafari E., Khandani B.K. (2018). Effect of Cinnamic acid and FOLFOX in diminishing side population and downregulating cancer stem cell markers in colon cancer cell line HT-29. Daru.

[169] Villota H., Santa-Gonzalez G.A., Uribe D., Henao I.C., Arroyave-Ospina J.C., Barrera-Causil C.J., Pedroza-Diaz J. (2022). Modulatory Effect of Chlorogenic Acid and Coffee Extracts on Wnt/beta-Catenin Pathway in Colorectal Cancer Cells. Nutrients.

[170] Aikins A.R., Birikorang P.A., Sabah S.B., Baffoe S.M., Tetteh J.K.A., Quarshie J.T., Quaye O. (2023). Caffeic Acid Inhibits Proliferation, Migration, and Stemness of DU-145 Prostate Cancer Cells. Nat. Prod. Commun..

[171] Zhang X., Lin D., Jiang R., Li H., Wan J., Li H. (2016). Ferulic acid exerts antitumor activity and inhibits metastasis in breast cancer cells by regulating epithelial to mesenchymal transition. Oncol. Rep..

[172] Zhang L., Wen X., Li M., Li S., Zhao H. (2018). Targeting cancer stem cells and signaling pathways by resveratrol and pterostilbene. Biofactors.

[173] Bhaskara V.K., Mittal B., Mysorekar V.V., Amaresh N., Simal-Gandara J. (2020). Resveratrol, cancer and cancer stem cells: A review on past to future. Curr. Res. Food Sci..

[174] Alrafas H.R., Busbee P.B., Chitrala K.N., Nagarkatti M., Nagarkatti P. (2020). Alterations in the Gut Microbiome and Suppression of Histone Deacetylases by Resveratrol Are Associated with Attenuation of Colonic Inflammation and Protection Against Colorectal Cancer. J. Clin. Med..

[175] Buhrmann C., Yazdi M., Popper B., Shayan P., Goel A., Aggarwal B.B., Shakibaei M. (2018). Resveratrol Chemosensitizes TNF-beta-Induced Survival of 5-FU-Treated Colorectal Cancer Cells. Nutrients.

[176] Reddivari L., Charepalli V., Radhakrishnan S., Vadde R., Elias R.J., Lambert J.D., Vanamala J.K. (2016). Grape compounds suppress colon cancer stem cells in vitro and in a rodent model of colon carcinogenesis. BMC Complement. Altern. Med..

[177] Nguyen A.V., Martinez M., Stamos M.J., Moyer M.P., Planutis K., Hope C., Holcombe R.F. (2009). Results of a phase I pilot clinical trial examining the effect of plant-derived resveratrol and grape powder on Wnt pathway target gene expression in colonic mucosa and colon cancer. Cancer Manag. Res..

[178] Fu Y., Chang H., Peng X., Bai Q., Yi L., Zhou Y., Zhu J., Mi M. (2014). Resveratrol inhibits breast cancer stem-like cells and induces autophagy via suppressing Wnt/beta-catenin signaling pathway. PLoS ONE.

[179] Pandey P.R., Okuda H., Watabe M., Pai S.K., Liu W., Kobayashi A., Xing F., Fukuda K., Hirota S., Sugai T. (2011). Resveratrol suppresses growth of cancer stem-like cells by inhibiting fatty acid synthase. Breast Cancer Res. Treat..

[180] Sun Y., Zhou Q.M., Lu Y.Y., Zhang H., Chen Q.L., Zhao M., Su S.B. (2019). Resveratrol Inhibits the Migration and Metastasis of MDA-MB-231 Human Breast Cancer by Reversing TGF-beta1-Induced Epithelial-Mesenchymal Transition. Molecules.

[181] Mak K.K., Wu A.T., Lee W.H., Chang T.C., Chiou J.F., Wang L.S., Wu C.H., Huang C.Y., Shieh Y.S., Chao T.Y. (2013). Pterostilbene, a bioactive component of blueberries, suppresses the generation of breast cancer stem cells within tumor microenvironment and metastasis via modulating NF-kappaB/microRNA 448 circuit. Mol. Nutr. Food Res..

[182] Rodriguez-Garcia C., Sanchez-Quesada C., Toledo E., Delgado-Rodriguez M., Gaforio J.J. (2019). Naturally Lignan-Rich Foods: A Dietary Tool for Health Promotion?. Molecules.

[183] Nguyen M., Osipo C. (2022). Targeting Breast Cancer Stem Cells Using Naturally Occurring Phytoestrogens. Int. J. Mol. Sci..

[184] Bayar İ., Akkoç S. (2024). A Mini Review on Components of Flax Seed and Their Effects on Breast Cancer. J. Agric. Sci..

[185] Jang W.Y., Kim M.Y., Cho J.Y. (2022). Antioxidant, Anti-Inflammatory, Anti-Menopausal, and Anti-Cancer Effects of Lignans and Their Metabolites. Int. J. Mol. Sci..

[186] Mahajan A., Sharma N., Ulhe A., Patil R., Hegde M., Mali A. (2024). From dietary lignans to cancer therapy: Integrative systems analysis of enterolactone’s molecular targets and signaling pathways in combatting cancer stem cells in triple-negative breast cancer. Food Biosci..

[187] Taibi A., Lin Z., Tsao R., Thompson L.U., Comelli E.M. (2019). Effects of Flaxseed and Its Components on Mammary Gland MiRNome: Identification of Potential Biomarkers to Prevent Breast Cancer Development. Nutrients.

[188] Memmola R., Petrillo A., Di Lorenzo S., Altuna S.C., Habeeb B.S., Soggiu A., Bonizzi L., Garrone O., Ghidini M. (2022). Correlation between Olive Oil Intake and Gut Microbiota in Colorectal Cancer Prevention. Nutrients.

[189] Vazquez-Martin A., Fernandez-Arroyo S., Cufi S., Oliveras-Ferraros C., Lozano-Sanchez J., Vellon L., Micol V., Joven J., Segura-Carretero A., Menendez J.A. (2012). Phenolic secoiridoids in extra virgin olive oil impede fibrogenic and oncogenic epithelial-to-mesenchymal transition: Extra virgin olive oil as a source of novel antiaging phytochemicals. Rejuvenation Res..

[190] Pistollato F., Giampieri F., Battino M. (2015). The use of plant-derived bioactive compounds to target cancer stem cells and modulate tumor microenvironment. Food Chem. Toxicol..

[191] Altundag-Erdogan O., Tutar R., Yuce E., Celebi-Saltik B. (2024). Targeting resistant breast cancer stem cells in a three-dimensional culture model with oleuropein encapsulated in methacrylated alginate microparticles. Daru.

[192] Cruz-Lozano M., Gonzalez-Gonzalez A., Marchal J.A., Munoz-Muela E., Molina M.P., Cara F.E., Brown A.M., Garcia-Rivas G., Hernandez-Brenes C., Lorente J.A. (2019). Hydroxytyrosol inhibits cancer stem cells and the metastatic capacity of triple-negative breast cancer cell lines by the simultaneous targeting of epithelial-to-mesenchymal transition, Wnt/beta-catenin and TGFbeta signaling pathways. Eur. J. Nutr..

[193] Leon-Gonzalez A.J., Saez-Martinez P., Jimenez-Vacas J.M., Herrero-Aguayo V., Montero-Hidalgo A.J., Gomez-Gomez E., Madrona A., Castano J.P., Espartero J.L., Gahete M.D. (2021). Comparative Cytotoxic Activity of Hydroxytyrosol and Its Semisynthetic Lipophilic Derivatives in Prostate Cancer Cells. Antioxidants.

[194] Bozorgi A., Khazaei S., Khademi A., Khazaei M. (2020). Natural and herbal compounds targeting breast cancer, a review based on cancer stem cells. Iran. J. Basic. Med. Sci..

[195] Mbese Z., Khwaza V., Aderibigbe B.A. (2019). Curcumin and Its Derivatives as Potential Therapeutic Agents in Prostate, Colon and Breast Cancers. Molecules.

[196] Ramasamy T.S., Ayob A.Z., Myint H.H., Thiagarajah S., Amini F. (2015). Targeting colorectal cancer stem cells using curcumin and curcumin analogues: Insights into the mechanism of the therapeutic efficacy. Cancer Cell Int..

[197] Su P., Yang Y., Wang G., Chen X., Ju Y. (2018). Curcumin attenuates resistance to irinotecan via induction of apoptosis of cancer stem cells in chemoresistant colon cancer cells. Int. J. Oncol..

[198] Shakibaei M., Buhrmann C., Kraehe P., Shayan P., Lueders C., Goel A. (2014). Curcumin chemosensitizes 5-fluorouracil resistant MMR-deficient human colon cancer cells in high density cultures. PLoS ONE.

[199] Wang K., Zhang T., Liu L., Wang X., Wu P., Chen Z., Ni C., Zhang J., Hu F., Huang J. (2012). Novel micelle formulation of curcumin for enhancing antitumor activity and inhibiting colorectal cancer stem cells. Int. J. Nanomed..

[200] Kantara C., O’Connell M., Sarkar S., Moya S., Ullrich R., Singh P. (2014). Curcumin promotes autophagic survival of a subset of colon cancer stem cells, which are ablated by DCLK1-siRNA. Cancer Res..

[201] James M.I., Iwuji C., Irving G., Karmokar A., Higgins J.A., Griffin-Teal N., Thomas A., Greaves P., Cai H., Patel S.R. (2015). Curcumin inhibits cancer stem cell phenotypes in ex vivo models of colorectal liver metastases, and is clinically safe and tolerable in combination with FOLFOX chemotherapy. Cancer Lett..

[202] Charpentier M.S., Whipple R.A., Vitolo M.I., Boggs A.E., Slovic J., Thompson K.N., Bhandary L., Martin S.S. (2014). Curcumin targets breast cancer stem-like cells with microtentacles that persist in mammospheres and promote reattachment. Cancer Res..

[203] Calaf G.M., Ponce-Cusi R., Abarca-Quinones J. (2018). Effect of curcumin on the cell surface markers CD44 and CD24 in breast cancer. Oncol. Rep..

[204] Li X., Wang X., Xie C., Zhu J., Meng Y., Chen Y., Li Y., Jiang Y., Yang X., Wang S. (2018). Sonic hedgehog and Wnt/beta-catenin pathways mediate curcumin inhibition of breast cancer stem cells. Anticancer Drugs.

[205] Chen W., Li L., Zhang X., Liang Y., Pu Z., Wang L., Mo J. (2017). Curcumin: A calixarene derivative micelle potentiates anti-breast cancer stem cells effects in xenografted, triple-negative breast cancer mouse models. Drug Deliv..

[206] Ros M., Riesco-Llach G., Polonio-Alcala E., Morla-Barcelo P.M., Ruiz-Martinez S., Feliu L., Planas M., Puig T. (2024). Inhibition of Cancer Stem-like Cells by Curcumin and Other Polyphenol Derivatives in MDA-MB-231 TNBC Cells. Int. J. Mol. Sci..

[207] Gallardo M., Kemmerling U., Aguayo F., Bleak T.C., Munoz J.P., Calaf G.M. (2020). Curcumin rescues breast cells from epithelial-mesenchymal transition and invasion induced by anti-miR-34a. Int. J. Oncol..

[208] Paramita P., Wardhani B.W., Wanandi S.I., Louisa M. (2018). Curcumin for the Prevention of Epithelial-Mesenchymal Transition in Endoxifen-Treated MCF-7 Breast Cancer Cel. Asian Pac. J. Cancer Prev..

[209] Sarighieh M.A., Montazeri V., Shadboorestan A., Ghahremani M.H., Ostad S.N. (2020). The Inhibitory Effect of Curcumin on Hypoxia Inducer Factors (Hifs) as a Regulatory Factor in the Growth of Tumor Cells in Breast Cancer Stem-Like Cells. Drug Res..

[210] Yang K., Liao Z., Wu Y., Li M., Guo T., Lin J., Li Y., Hu C. (2020). Curcumin and Glu-GNPs Induce Radiosensitivity against Breast Cancer Stem-Like Cells. Biomed. Res. Int..

[211] Zhou Q., Ye M., Lu Y., Zhang H., Chen Q., Huang S., Su S. (2015). Curcumin Improves the Tumoricidal Effect of Mitomycin C by Suppressing ABCG2 Expression in Stem Cell-Like Breast Cancer Cells. PLoS ONE.

[212] Ma Q., Qian W., Tao W., Zhou Y., Xue B. (2019). Delivery Of Curcumin Nanoliposomes Using Surface Modified With CD133 Aptamers For Prostate Cancer. Drug Des. Devel Ther..

[213] Panahizadeh R., Vatankhah M.A., Jeddi F., Arabzadeh A., Nejati-Koshki K., Salimnejad R., Najafzadeh N. (2023). Cytotoxicity of curcumin against CD44(+/−) prostate cancer cells: Roles of miR-383 and miR-708. Avicenna J. Phytomed..

[214] Khazbak A.E.-R.A., Salem S.M., Rady H.H., Bashandy M.A. (2023). Regulation of Breast Cancer Stem Cells Associated-miRNAs by Ginger and Persimmon Extracts. Egypt. Acad. J. Biol. Sci. C Physiol. Mol. Biol..

[215] Sp N., Kang D.Y., Lee J.M., Bae S.W., Jang K.J. (2021). Potential Antitumor Effects of 6-Gingerol in p53-Dependent Mitochondrial Apoptosis and Inhibition of Tumor Sphere Formation in Breast Cancer Cells. Int. J. Mol. Sci..

[216] Visan S., Soritau O., Tatomir C., Baldasici O., Balacescu L., Balacescu O., Muntean P., Gherasim C., Pintea A. (2023). The Bioactive Properties of Carotenoids from Lipophilic Sea buckthorn Extract (*Hippophae rhamnoides* L.) in Breast Cancer Cell Lines. Molecules.

[217] Lee W.S., Shin J.S., Jang S.Y., Chung K.S., Kim S.D., Cho C.W., Hong H.D., Rhee Y.K., Lee K.T. (2024). Anti-Metastatic Effects of Standardized Polysaccharide Fraction from Diospyros kaki Leaves via GSK3beta/beta-Catenin and JNK Inactivation in Human Colon Cancer Cells. Polymers.

[218] Artusa V., Calabrone L., Mortara L., Peri F., Bruno A. (2023). Microbiota-Derived Natural Products Targeting Cancer Stem Cells: Inside the Gut Pharma Factory. Int. J. Mol. Sci..

[219] Wan L., Tan H.L., Thomas-Ahner J.M., Pearl D.K., Erdman J.W., Moran N.E., Clinton S.K. (2014). Dietary tomato and lycopene impact androgen signaling- and carcinogenesis-related gene expression during early TRAMP prostate carcinogenesis. Cancer Prev. Res..

[220] Chen X., Yang G., Liu M., Quan Z., Wang L., Luo C., Wu X., Zheng Y. (2022). Lycopene enhances the sensitivity of castration-resistant prostate cancer to enzalutamide through the AKT/EZH2/ androgen receptor signaling pathway. Biochem. Biophys. Res. Commun..

[221] Kim D., Kim Y., Kim Y. (2019). Effects of beta-carotene on Expression of Selected MicroRNAs, Histone Acetylation, and DNA Methylation in Colon Cancer Stem Cells. J. Cancer Prev..

[222] Lee K.E., Kwon M., Kim Y.S., Kim Y., Chung M.G., Heo S.C., Kim Y. (2022). beta-carotene regulates cancer stemness in colon cancer in vivo and in vitro. Nutr. Res. Pract..

[223] Lee J., Heo S.C., Kim Y. (2024). Combination of oxaliplatin and beta-carotene suppresses colorectal cancer by regulating cell cycle, apoptosis, and cancer stemness in vitro. Nutr. Res. Pract..

[224] Carotenuto P., Pecoraro A., Brignola C., Barbato A., Franco B., Longobardi G., Conte C., Quaglia F., Russo G., Russo A. (2023). Combining beta-Carotene with 5-FU via Polymeric Nanoparticles as a Novel Therapeutic Strategy to Overcome uL3-Mediated Chemoresistance in p53-Deleted Colorectal Cancer Cells. Mol. Pharm..

[225] Li Y., Zhang Y., Liu X., Wang M., Wang P., Yang J., Zhang S. (2018). Lutein inhibits proliferation, invasion and migration of hypoxic breast cancer cells via downregulation of HES1. Int. J. Oncol..

[226] Ahn Y.T., Kim M.S., Kim Y.S., An W.G. (2020). Astaxanthin Reduces Stemness Markers in BT20 and T47D Breast Cancer Stem Cells by Inhibiting Expression of Pontin and Mutant p53. Mar. Drugs.

[227] de la Mare J.A., Sterrenberg J.N., Sukhthankar M.G., Chiwakata M.T., Beukes D.R., Blatch G.L., Edkins A.L. (2013). Assessment of potential anti-cancer stem cell activity of marine algal compounds using an in vitro mammosphere assay. Cancer Cell Int..

[228] Gullu N., Kobelt D., Brim H., Rahman S., Timm L., Smith J., Soleimani A., Di Marco S., Bisti S., Ashktorab H. (2020). Saffron Crudes and Compounds Restrict MACC1-Dependent Cell Proliferation and Migration of Colorectal Cancer Cells. Cells.

[229] Ghorbanzadeh V., Hassan Aljaf K.A., Wasman H.M., Dariushnejad H. (2024). Crocin inhibit the metastasis of MDA-MB-231 cell line by suppressing epithelial to mesenchymal transition through WNT/beta-catenin signalling pathway. Ann. Med. Surg..

[230] Khan M., Hearn K., Parry C., Rashid M., Brim H., Ashktorab H., Kwabi-Addo B. (2023). Mechanism of Antitumor Effects of Saffron in Human Prostate Cancer Cells. Nutrients.

[231] Yang X., Liang B., Zhang L., Zhang M., Ma M., Qing L., Yang H., Huang G., Zhao J. (2024). Ursolic acid inhibits the proliferation of triple-negative breast cancer stem-like cells through NRF2-mediated ferroptosis. Oncol. Rep..

[232] Kim G.D. (2021). Ursolic Acid Decreases the Proliferation of MCF-7 Cell-Derived Breast Cancer Stem-Like Cells by Modulating the ERK and PI3K/AKT Signaling Pathways. Prev. Nutr. Food Sci..

[233] Liao W.L., Liu Y.F., Ying T.H., Shieh J.C., Hung Y.T., Lee H.J., Shen C.Y., Cheng C.W. (2022). Inhibitory Effects of Ursolic Acid on the Stemness and Progression of Human Breast Cancer Cells by Modulating Argonaute-2. Int. J. Mol. Sci..

[234] Chen R., Wu Y., Wang F., Zhou J., Zhuang H., LI W. (2024). Oleanolic acid inhibits colon cancer cell stemness and reverses chemoresistance by suppressing JAK2/STAT3 signaling pathway. Biocell.

[235] Wojdylo A., Turkiewicz I.P., Tkacz K., Nowicka P., Bobak L. (2022). Nuts as functional foods: Variation of nutritional and phytochemical profiles and their in vitro bioactive properties. Food Chem. X.

[236] Aggarwal V., Kashyap D., Sak K., Tuli H.S., Jain A., Chaudhary A., Garg V.K., Sethi G., Yerer M.B. (2019). Molecular Mechanisms of Action of Tocotrienols in Cancer: Recent Trends and Advancements. Int. J. Mol. Sci..

[237] Gu W., Prasadam I., Yu M., Zhang F., Ling P., Xiao Y., Yu C. (2015). Gamma tocotrienol targets tyrosine phosphatase SHP2 in mammospheres resulting in cell death through RAS/ERK pathway. BMC Cancer.

[238] Ahmed R.A., Alawin O.A., Sylvester P.W. (2016). gamma-Tocotrienol reversal of epithelial-to-mesenchymal transition in human breast cancer cells is associated with inhibition of canonical Wnt signalling. Cell Prolif..

[239] Luk S.U., Yap W.N., Chiu Y.T., Lee D.T., Ma S., Lee T.K., Vasireddy R.S., Wong Y.C., Ching Y.P., Nelson C. (2011). Gamma-tocotrienol as an effective agent in targeting prostate cancer stem cell-like population. Int. J. Cancer.

[240] Kaneko S., Sato C., Shiozawa N., Sato A., Sato H., Virgona N., Yano T. (2018). Suppressive Effect of Delta-Tocotrienol on Hypoxia Adaptation of Prostate Cancer Stem-like Cells. Anticancer. Res..

[241] Dacrema M., Ali A., Ullah H., Khan A., Di Minno A., Xiao J., Martins A.M.C., Daglia M. (2022). Spice-Derived Bioactive Compounds Confer Colorectal Cancer Prevention via Modulation of Gut Microbiota. Cancers.

[242] Feitelson M.A., Arzumanyan A., Medhat A., Spector I. (2023). Short-chain fatty acids in cancer pathogenesis. Cancer Metastasis Rev..

[243] Shim Y., Song J.M. (2015). Quantum dot nanoprobe-based high-content monitoring of notch pathway inhibition of breast cancer stem cell by capsaicin. Mol. Cell Probes.

[244] Zhu M., Yu X., Zheng Z., Huang J., Yang X., Shi H. (2020). Capsaicin suppressed activity of prostate cancer stem cells by inhibition of Wnt/beta-catenin pathway. Phytother. Res..

[245] Hakeem A.N., El-Kersh D.M., Hammam O., Elhosseiny A., Zaki A., Kamel K., Yasser L., Barsom M., Ahmed M., Gamal M. (2024). Piperine enhances doxorubicin sensitivity in triple-negative breast cancer by targeting the PI3K/Akt/mTOR pathway and cancer stem cells. Sci. Rep..

[246] Ly H.T., Tran P.T., Le B.V., Nguyen T.M., Nguyen T.H.L., Nguyen T.T., Dao A.H., Le V.M., Kang K.W., Do T.H. (2024). Standardized extract and its compounds from fruits of Piper longum suppress MDA-MB-231 cancer stem cells via down-regulation of intracellular signals. S. Afr. J. Bot..

[247] Talib W.H., Baban M.M., Azzam A.O., Issa J.J., Ali A.Y., AlSuwais A.K., Allala S., Al Kury L.T. (2024). Allicin and Cancer Hallmarks. Molecules.

[248] Brugnoli F., Tedeschi P., Grassilli S., Maietti A., Brandolini V., Bertagnolo V. (2021). Ethanol-based garlic extract prevents malignant evolution of non-invasive breast tumor cells induced by moderate hypoxia. Biomed. Pharmacother..

[249] Kim S.H., Kaschula C.H., Priedigkeit N., Lee A.V., Singh S.V. (2016). Forkhead Box Q1 Is a Novel Target of Breast Cancer Stem Cell Inhibition by Diallyl Trisulfide. J. Biol. Chem..

[250] Li X., Meng Y., Xie C., Zhu J., Wang X., Li Y., Geng S., Wu J., Zhong C., Li M. (2018). Diallyl Trisulfide inhibits breast cancer stem cells via suppression of Wnt/beta-catenin pathway. J. Cell Biochem..

[251] Xie X., Huang X., Tang H., Ye F., Yang L., Guo X., Tian Z., Xie X., Peng C., Xie X. (2018). Diallyl Disulfide Inhibits Breast Cancer Stem Cell Progression and Glucose Metabolism by Targeting CD44/PKM2/AMPK Signaling. Curr. Cancer Drug Targets.

[252] Shoaib S., Ansari M.A., Ghazwani M., Hani U., Jamous Y.F., Alali Z., Wahab S., Ahmad W., Weir S.A., Alomary M.N. (2023). Prospective Epigenetic Actions of Organo-Sulfur Compounds against Cancer: Perspectives and Molecular Mechanisms. Cancers.

[253] Miekus N., Marszalek K., Podlacha M., Iqbal A., Puchalski C., Swiergiel A.H. (2020). Health Benefits of Plant-Derived Sulfur Compounds, Glucosinolates, and Organosulfur Compounds. Molecules.

[254] Shin J.M., Lim E., Cho Y.S., Nho C.W. (2021). Cancer-preventive effect of phenethyl isothiocyanate through tumor microenvironment regulation in a colorectal cancer stem cell xenograft model. Phytomedicine.

[255] Zhang T., Zhang W., Hao M. (2021). Phenethyl isothiocyanate reduces breast cancer stem cell-like properties by epigenetic reactivation of CDH1. Oncol. Rep..

[256] Pereira L.P., Silva P., Duarte M., Rodrigues L., Duarte C.M., Albuquerque C., Serra A.T. (2017). Targeting Colorectal Cancer Proliferation, Stemness and Metastatic Potential Using Brassicaceae Extracts Enriched in Isothiocyanates: A 3D Cell Model-Based Study. Nutrients.

[257] Li Y., Zhang T., Korkaya H., Liu S., Lee H.F., Newman B., Yu Y., Clouthier S.G., Schwartz S.J., Wicha M.S. (2010). Sulforaphane, a dietary component of broccoli/broccoli sprouts, inhibits breast cancer stem cells. Clin. Cancer Res..

[258] Simoes B.M., Santiago-Gomez A., Chiodo C., Moreira T., Conole D., Lovell S., Alferez D., Eyre R., Spence K., Sarmiento-Castro A. (2020). Targeting STAT3 signaling using stabilised sulforaphane (SFX-01) inhibits endocrine resistant stem-like cells in ER-positive breast cancer. Oncogene.

[259] Burnett J.P., Lim G., Li Y., Shah R.B., Lim R., Paholak H.J., McDermott S.P., Sun L., Tsume Y., Bai S. (2017). Sulforaphane enhances the anticancer activity of taxanes against triple negative breast cancer by killing cancer stem cells. Cancer Lett..

[260] Vyas A.R., Moura M.B., Hahm E.R., Singh K.B., Singh S.V. (2016). Sulforaphane Inhibits c-Myc-Mediated Prostate Cancer Stem-Like Traits. J. Cell Biochem..

[261] Labsch S., Liu L., Bauer N., Zhang Y., Aleksandrowicz E., Gladkich J., Schonsiegel F., Herr I. (2014). Sulforaphane and TRAIL induce a synergistic elimination of advanced prostate cancer stem-like cells. Int. J. Oncol..

[262] Kansal S., Negi A.K., Agnihotri N. (2012). n-3 PUFAs as Modulators of Stem Cells in Prevention of Colorectal Cancer. Curr. Color. Cancer Rep..

[263] Goncalves B., Pinto T., Aires A., Morais M.C., Bacelar E., Anjos R., Ferreira-Cardoso J., Oliveira I., Vilela A., Cosme F. (2023). Composition of Nuts and Their Potential Health Benefits-An Overview. Foods.

[264] De Carlo F., Witte T.R., Hardman W.E., Claudio P.P. (2013). Omega-3 eicosapentaenoic acid decreases CD133 colon cancer stem-like cell marker expression while increasing sensitivity to chemotherapy. PLoS ONE.

[265] Yang T., Fang S., Zhang H.X., Xu L.X., Zhang Z.Q., Yuan K.T., Xue C.L., Yu H.L., Zhang S., Li Y.F. (2013). N-3 PUFAs have antiproliferative and apoptotic effects on human colorectal cancer stem-like cells in vitro. J. Nutr. Biochem..

[266] Sam M.R., Ahangar P., Nejati V., Habibian R. (2016). Treatment of LS174T colorectal cancer stem-like cells with n-3 PUFAs induces growth suppression through inhibition of survivin expression and induction of caspase-3 activation. Cell Oncol..

[267] Sam M.R., Tavakoli-Mehr M., Safaralizadeh R. (2018). Omega-3 fatty acid DHA modulates p53, survivin, and microRNA-16-1 expression in KRAS-mutant colorectal cancer stem-like cells. Genes. Nutr..

[268] Volpato M., Ingram N., Perry S.L., Spencer J., Race A.D., Marshall C., Hutchinson J.M., Nicolaou A., Loadman P.M., Coletta P.L. (2021). Cyclooxygenase activity mediates colorectal cancer cell resistance to the omega-3 polyunsaturated fatty acid eicosapentaenoic acid. Cancer Chemother. Pharmacol..

[269] Li H., Feng Z., He M.L. (2020). Lipid metabolism alteration contributes to and maintains the properties of cancer stem cells. Theranostics.

[270] Kim J.S., Kim D.K., Moon J.Y., Lee M.-Y., Cho S.K. (2024). Oleic acid inhibits the migration and invasion of breast cancer cells with stemness characteristics through oxidative stress-mediated attenuation of the FAK/AKT/NF-κB pathway. J. Funct. Foods.

[271] Kim H.S., Jung M., Choi S.K., Moon W.K., Kim S.J. (2016). Different Biological Action of Oleic Acid in ALDHhigh and ALDHlow Subpopulations Separated from Ductal Carcinoma In Situ of Breast Cancer. PLoS ONE.

[272] Hwang S.H., Yang Y., Jung J.H., Kim Y. (2022). Oleic acid from cancer-associated fibroblast promotes cancer cell stemness by stearoyl-CoA desaturase under glucose-deficient condition. Cancer Cell Int..

[273] Choi S., Yoo Y.J., Kim H., Lee H., Chung H., Nam M.H., Moon J.Y., Lee H.S., Yoon S., Kim W.Y. (2019). Clinical and biochemical relevance of monounsaturated fatty acid metabolism targeting strategy for cancer stem cell elimination in colon cancer. Biochem. Biophys. Res. Commun..

[274] Choudhury P., Barua A., Roy A., Pattanayak R., Bhattacharyya M., Saha P. (2020). Eugenol restricts Cancer Stem Cell population by degradation of beta-catenin via N-terminal Ser37 phosphorylation-an in vivo and in vitro experimental evaluation. Chem. Biol. Interact..

[275] Islam S.S., Al-Sharif I., Sultan A., Al-Mazrou A., Remmal A., Aboussekhra A. (2018). Eugenol potentiates cisplatin anti-cancer activity through inhibition of ALDH-positive breast cancer stem cells and the NF-kappaB signaling pathway. Mol. Carcinog..

[276] Wu C.E., Zhuang Y.W., Zhou J.Y., Liu S.L., Wang R.P., Shu P. (2019). Cinnamaldehyde enhances apoptotic effect of oxaliplatin and reverses epithelial-mesenchymal transition and stemnness in hypoxic colorectal cancer cells. Exp. Cell Res..

[277] Ismail I.A., Kang H.S., Lee H.J., Chang H., Yun J., Lee C.W., Kim N.H., Kim H.S., Yook J.I., Hong S.H. (2013). 2-Hydroxycinnamaldehyde inhibits the epithelial-mesenchymal transition in breast cancer cells. Breast Cancer Res. Treat..

[278] Fatfat Z., Fatfat M., Gali-Muhtasib H. (2021). Therapeutic potential of thymoquinone in combination therapy against cancer and cancer stem cells. World J. Clin. Oncol..

[279] Karim S., Burzangi A.S., Ahmad A., Siddiqui N.A., Ibrahim I.M., Sharma P., Abualsunun W.A., Gabr G.A. (2022). PI3K-AKT Pathway Modulation by Thymoquinone Limits Tumor Growth and Glycolytic Metabolism in Colorectal Cancer. Int. J. Mol. Sci..

[280] Bashmail H.A., Alamoudi A.A., Noorwali A., Hegazy G.A., Ajabnoor G.M., Al-Abd A.M. (2020). Thymoquinone Enhances Paclitaxel Anti-Breast Cancer Activity via Inhibiting Tumor-Associated Stem Cells Despite Apparent Mathematical Antagonism. Molecules.

[281] Bawadud Rs B., Alkhatib M.H., Gashlan Hm G. (2020). Combination of Docetaxel with Thymoquinone in Nanoemulsion Impedes the Migration of Breast Cancer Stem Cells. Int. J. Pharm. Investig..

[282] Haiaty S., Rashidi M.R., Akbarzadeh M., Bazmani A., Mostafazadeh M., Nikanfar S., Zibaei Z., Rahbarghazi R., Nouri M. (2021). Thymoquinone inhibited vasculogenic capacity and promoted mesenchymal-epithelial transition of human breast cancer stem cells. BMC Complement. Med. Ther..

[283] Ballout F., Monzer A., Fatfat M., Ouweini H.E., Jaffa M.A., Abdel-Samad R., Darwiche N., Abou-Kheir W., Gali-Muhtasib H. (2020). Thymoquinone induces apoptosis and DNA damage in 5-Fluorouracil-resistant colorectal cancer stem/progenitor cells. Oncotarget.

[284] Teferra T.F. (2021). Possible actions of inulin as prebiotic polysaccharide: A review. Food Front..

[285] Sheng W., Ji G., Zhang L. (2023). Immunomodulatory effects of inulin and its intestinal metabolites. Front. Immunol..

[286] Zhang W., An Y., Qin X., Wu X., Wang X., Hou H., Song X., Liu T., Wang B., Huang X. (2021). Gut Microbiota-Derived Metabolites in Colorectal Cancer: The Bad and the Challenges. Front. Oncol..

[287] He Y., Ling Y., Zhang Z., Mertens R.T., Cao Q., Xu X., Guo K., Shi Q., Zhang X., Huo L. (2023). Butyrate reverses ferroptosis resistance in colorectal cancer by inducing c-Fos-dependent xCT suppression. Redox Biol..

[288] Takebe N., Harris P.J., Warren R.Q., Ivy S.P. (2011). Targeting cancer stem cells by inhibiting Wnt, Notch, and Hedgehog pathways. Nat. Rev. Clin. Oncol..

[289] Zheng Y., Wang L., Yin L., Yao Z., Tong R., Xue J., Lu Y. (2022). Lung Cancer Stem Cell Markers as Therapeutic Targets: An Update on Signaling Pathways and Therapies. Front. Oncol..

[290] Zeng Z., Fu M., Hu Y., Wei Y., Wei X., Luo M. (2023). Regulation and signaling pathways in cancer stem cells: Implications for targeted therapy for cancer. Mol. Cancer.

[291] Venkatesh V., Nataraj R., Thangaraj G.S., Karthikeyan M., Gnanasekaran A., Kaginelli S.B., Kuppanna G., Kallappa C.G., Basalingappa K.M. (2018). Targeting Notch signalling pathway of cancer stem cells. Stem Cell Investig..

[292] Liu J., Xiao Q., Xiao J., Niu C., Li Y., Zhang X., Zhou Z., Shu G., Yin G. (2022). Wnt/beta-catenin signalling: Function, biological mechanisms, and therapeutic opportunities. Signal Transduct. Target. Ther..

[293] Yang Y., Li X., Wang T., Guo Q., Xi T., Zheng L. (2020). Emerging agents that target signaling pathways in cancer stem cells. J. Hematol. Oncol..

[294] Liu T., Zhang L., Joo D., Sun S.C. (2017). NF-kappaB signaling in inflammation. Signal Transduct. Target. Ther..

[295] Rinkenbaugh A.L., Baldwin A.S. (2016). The NF-kappaB Pathway and Cancer Stem Cells. Cells.

[296] Panwar V., Singh A., Bhatt M., Tonk R.K., Azizov S., Raza A.S., Sengupta S., Kumar D., Garg M. (2023). Multifaceted role of mTOR (mammalian target of rapamycin) signaling pathway in human health and disease. Signal Transduct. Target. Ther..

[297] Galoczova M., Coates P., Vojtesek B. (2018). STAT3, stem cells, cancer stem cells and p63. Cell Mol. Biol. Lett..

[298] Hu X., Li J., Fu M., Zhao X., Wang W. (2021). The JAK/STAT signaling pathway: From bench to clinic. Signal Transduct. Target. Ther..

[299] Hayward N.J., McDougall G.J., Farag S., Allwood J.W., Austin C., Campbell F., Horgan G., Ranawana V. (2019). Cinnamon Shows Antidiabetic Properties that Are Species-Specific: Effects on Enzyme Activity Inhibition and Starch Digestion. Plant Foods Hum. Nutr..

[300] Yang L., Shi P., Zhao G., Xu J., Peng W., Zhang J., Zhang G., Wang X., Dong Z., Chen F. (2020). Targeting cancer stem cell pathways for cancer therapy. Signal Transduct. Target. Ther..

[301] Kim D.H., Xing T., Yang Z., Dudek R., Lu Q., Chen Y.H. (2017). Epithelial Mesenchymal Transition in Embryonic Development, Tissue Repair and Cancer: A Comprehensive Overview. J. Clin. Med..

[302] Shibue T., Weinberg R.A. (2017). EMT, CSCs, and drug resistance: The mechanistic link and clinical implications. Nat. Rev. Clin. Oncol..

[303] Tanabe S., Quader S., Cabral H., Ono R. (2020). Interplay of EMT and CSC in Cancer and the Potential Therapeutic Strategies. Front. Pharmacol..

[304] Cho R.W., Wang X., Diehn M., Shedden K., Chen G.Y., Sherlock G., Gurney A., Lewicki J., Clarke M.F. (2008). Isolation and molecular characterization of cancer stem cells in MMTV-Wnt-1 murine breast tumors. Stem Cells.

[305] Chu X., Tian W., Ning J., Xiao G., Zhou Y., Wang Z., Zhai Z., Tanzhu G., Yang J., Zhou R. (2024). Cancer stem cells: Advances in knowledge and implications for cancer therapy. Signal Transduct. Target. Ther..

[306] Jogi A., Vaapil M., Johansson M., Pahlman S. (2012). Cancer cell differentiation heterogeneity and aggressive behavior in solid tumors. Ups. J. Med. Sci..

[307] Jelski W., Mroczko B. (2020). Biochemical Markers of Colorectal Cancer—Present and Future. Cancer Manag. Res..

[308] Gao Y., Wang J., Zhou Y., Sheng S., Qian S.Y., Huo X. (2018). Evaluation of Serum CEA, CA19-9, CA72-4, CA125 and Ferritin as Diagnostic Markers and Factors of Clinical Parameters for Colorectal Cancer. Sci. Rep..

[309] Bratt O., Lilja H. (2015). Serum markers in prostate cancer detection. Curr. Opin. Urol..

[310] Kabel A.M. (2017). Tumor markers of breast cancer: New prospectives. J. Oncol. Sci..

[311] Kuipers E.J., Grady W.M., Lieberman D., Seufferlein T., Sung J.J., Boelens P.G., van de Velde C.J., Watanabe T. (2015). Colorectal cancer. Nat. Rev. Dis. Primers.

[312] Arena E.A., Bilchik A.J. (2013). What is the optimal means of staging colon cancer?. Adv. Surg..

[313] da Luz F.A.C., Araujo B.J., de Araujo R.A. (2022). The current staging and classification systems of breast cancer and their pitfalls: Is it possible to integrate the complexity of this neoplasm into a unified staging system?. Crit. Rev. Oncol. Hematol..

[314] Koh J., Kim M.J. (2019). Introduction of a New Staging System of Breast Cancer for Radiologists: An Emphasis on the Prognostic Stage. Korean J. Radiol..

[315] Sathianathen N.J., Konety B.R., Crook J., Saad F., Lawrentschuk N. (2018). Landmarks in prostate cancer. Nat. Rev. Urol..

[316] de Thé H. (2018). Differentiation therapy revisited. Nat. Rev. Cancer.

[317] Lo-Coco F., Avvisati G., Vignetti M., Thiede C., Orlando S.M., Iacobelli S., Ferrara F., Fazi P., Cicconi L., Di Bona E. (2013). Retinoic acid and arsenic trioxide for acute promyelocytic leukemia. N. Engl. J. Med..

[318] Greil R., Greil-Ressler S., Weiss L., Schonlieb C., Magnes T., Radl B., Bolger G.T., Vcelar B., Sordillo P.P. (2018). A phase 1 dose-escalation study on the safety, tolerability and activity of liposomal curcumin (Lipocurc^TM^) in patients with locally advanced or metastatic cancer. Cancer Chemother. Pharmacol..

[319] Bayet-Robert M., Kwiatkowski F., Leheurteur M., Gachon F., Planchat E., Abrial C., Mouret-Reynier M.A., Durando X., Barthomeuf C., Chollet P. (2010). Phase I dose escalation trial of docetaxel plus curcumin in patients with advanced and metastatic breast cancer. Cancer Biol. Ther..

[320] Kucuk O., Sarkar F.H., Djuric Z., Sakr W., Pollak M.N., Khachik F., Banerjee M., Bertram J.S., Wood D.P. (2002). Effects of lycopene supplementation in patients with localized prostate cancer. Exp. Biol. Med..

[321] Zhuang E., Uchio E., Lilly M., Zi X., Fruehauf J.P. (2021). A phase II study of docetaxel plus lycopene in metastatic castrate resistant prostate cancer. Biomed. Pharmacother..

[322] Lilly M.B., Wu C., Ke Y., Chen W.P., Soloff A.C., Armeson K., Yokoyama N.N., Li X., Song L., Yuan Y. (2024). A phase I study of docetaxel plus synthetic lycopene in metastatic prostate cancer patients. Clin. Transl. Med..

[323] Ma C.J., Wu J.M., Tsai H.L., Huang C.W., Lu C.Y., Sun L.C., Shih Y.L., Chen C.W., Chuang J.F., Wu M.H. (2015). Prospective double-blind randomized study on the efficacy and safety of an n-3 fatty acid enriched intravenous fat emulsion in postsurgical gastric and colorectal cancer patients. Nutr. J..

[324] Bakker N., van den Helder R.S., Stoutjesdijk E., van Pelt J., Houdijk A.P.J. (2020). Effects of perioperative intravenous omega-3 fatty acids in colon cancer patients: A randomized, double-blind, placebo-controlled clinical trial. Am. J. Clin. Nutr..

[325] Anoushirvani A.A., Poorsaadat L., Aghabozorgi R., Kasravi M. (2018). Comparison of the Effects of Omega 3 and Vitamin E on Palcitaxel-Induced Peripheral Neuropathy. Open Access Maced. J. Med. Sci..

[326] Tawfik B., Dayao Z.R., Brown-Glaberman U.A., Pankratz V.S., Lafky J.M., Loprinzi C.L., Barton D.L. (2023). A pilot randomized, placebo-controlled, double-blind study of omega-3 fatty acids to prevent paclitaxel-associated acute pain syndrome in breast cancer patients: Alliance A22_Pilot2. Support. Care Cancer.

[327] Crockett S.D., Barry E.L., Mott L.A., Ahnen D.J., Robertson D.J., Anderson J.C., Wallace K., Burke C.A., Bresalier R.S., Figueiredo J.C. (2019). Calcium and vitamin D supplementation and increased risk of serrated polyps: Results from a randomised clinical trial. Gut.

[328] Going C.C., Alexandrova L., Lau K., Yeh C.Y., Feldman D., Pitteri S.J. (2018). Vitamin D supplementation decreases serum 27-hydroxycholesterol in a pilot breast cancer trial. Breast Cancer Res. Treat..

[329] Schwartz G.G., Hall M.C., Stindt D., Patton S., Lovato J., Torti F.M. (2005). Phase I/II study of 19-nor-1alpha-25-dihydroxyvitamin D2 (paricalcitol) in advanced, androgen-insensitive prostate cancer. Clin. Cancer Res..

[330] Hardiman G., Savage S.J., Hazard E.S., Wilson R.C., Courtney S.M., Smith M.T., Hollis B.W., Halbert C.H., Gattoni-Celli S. (2016). Systems analysis of the prostate transcriptome in African-American men compared with European-American men. Pharmacogenomics.

[331] Marshall D.T., Savage S.J., Garrett-Mayer E., Keane T.E., Hollis B.W., Horst R.L., Ambrose L.H., Kindy M.S., Gattoni-Celli S. (2012). Vitamin D3 supplementation at 4000 international units per day for one year results in a decrease of positive cores at repeat biopsy in subjects with low-risk prostate cancer under active surveillance. J. Clin. Endocrinol. Metab..

[332] Recchia F., Saggio G., Cesta A., Candeloro G., Di Blasio A., Amiconi G., Lombardo M., Nuzzo A., Lalli A., Alesse E. (2007). Phase II study of interleukin-2 and 13-cis-retinoic acid as maintenance therapy in metastatic colorectal cancer. Cancer Immunol. Immunother..

[333] Bryan M., Pulte E.D., Toomey K.C., Pliner L., Pavlick A.C., Saunders T., Wieder R. (2011). A pilot phase II trial of all-trans retinoic acid (Vesanoid) and paclitaxel (Taxol) in patients with recurrent or metastatic breast cancer. Investig. New Drugs.

[334] Ferrari A.C., Stone N., Stock R., Bednar M., Esseesse I., Singh H., Baldwin Y., Mandeli J. (2002). 13-cis retinoic acid and complete androgen blockade in advanced hormone-naive prostate cancer patients: Report of a phase II randomized study. J. Clin. Oncol..

